# Vibrational Circular Dichroism from DFT Molecular Dynamics: The AWV Method

**DOI:** 10.1021/acs.jctc.2c00736

**Published:** 2022-09-16

**Authors:** Daria Ruth Galimberti

**Affiliations:** † Institute for Molecules and Materials, Radboud University, Heyendaalseweg 135, 6525 AJ Nijmegen, The Netherlands

## Abstract

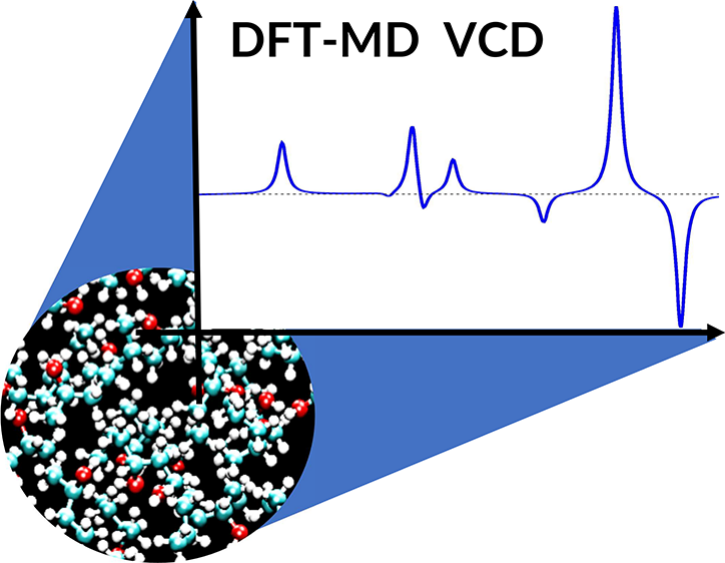

The paper illustrates the Activity Weighted Velocities (AWV) methodology to compute Vibrational Circular Dichroism (VCD) anharmonic spectra from Density Functional Theory (DFT) molecular dynamics. AWV calculates the spectra by the Fourier Transform of the time correlation functions of velocities, weighted by specific observables: the Atomic Polar Tensors (APTs) and the Atomic Axial Tensors (AATs). Indeed, AWV shows to correctly reproduce the experimental spectra for systems in the gas and liquid phases, both in the case of weakly and strongly interacting systems. The comparison with the experimental spectra is striking especially in the fingerprint region, as demonstrated by the three benchmark systems discussed: (1*S*)-Fenchone in the gas phase, (*S*)-(−)-Propylene oxide in the liquid phase, and (*R*)-(−)-2-butanol in the liquid phase. The time evolution of APTs and AATs can be adequately described by a linear combination of the tensors of a small set of appropriate reference structures, strongly reducing the computational cost without compromising accuracy. Additionally, AWV allows the partition of the spectral signal in its molecular components without any expensive postprocessing and any localization of the charge density or the wave function.

## 1 Introduction

Vibrational Circular Dichroism (VCD) is a chiral vibrational spectroscopy sensitive to a target system’s molecular and supramolecular chirality. Information on the conformation and the intra- and intermolecular interactions can be obtained.^1−6^ The successful application of this spectroscopic technique to the study of complex phenomena, such as the aggregation of fibrils or the structural aspects of proteins,^3−6^ of natural products,^7−9^ host–guest interactions and encapsulation processes,^10,11^ of liquid crystals,^12,13^ and catalytic nanoparticles,^14−17^ have demonstrated its power and explain the momentum VCD is gaining in these last years.

Due to the variety and complexity of the factors interplaying in determining the spectral shape, the interpretation of spectroscopic data based on experimental measurements alone can lead to uncertainties and ambiguities. In this respect, theoretical calculations are a powerful tool for analyzing the connection between structure and spectroscopic marker bands.^18−21^

Standard quantum chemistry software packages^20,22−24^ can nowadays compute VCD *static* spectra (i.e., of equilibrium structures) in the double harmonic approximation. Efforts have been made to go beyond the harmonic approximation, and approaches have been implemented to account for anharmonic effects.^25^ As powerful as it is, this *static* approach cannot be applied straightforwardly in the case of a liquid phase, especially when strong intermolecular interactions are expected. Multiple intra- and intermolecular conformations contribute to the final spectrum and must be considered. A common strategy is to run first Molecular Dynamics (MD) simulations to correctly sample the intra- and intermolecular conformational space in the liquid phase; in a second step, statistical clustering methods are applied to extract a set representative set of local structures/clusters from the trajectories.^19,26−28^ Finally, the spectrum is obtained as the weighted average of the *static* spectra on this selected set. One delicate point of this protocol that must be carefully considered is how to choose the variables to identify the relevant conformers in the clustering step univocally. Particular difficulties are encountered when a significant modulation of the band shapes is determined by limited structure variations.^19^

In the recent literature, a different approach has been proposed to find a way to bypass these issues: the time correlation formalism.^21,25,29,30^ Within the time-correlation function formalism, an MD simulation is run. Then, the VCD absorption spectrum of an isotropic system is computed from the time cross-correlation function of the system’s electric and magnetic dipole moment.^21,31^ Because the VCD response is directly calculated from the MD trajectories, a single run samples the contributions of different configurations. No harmonic approximation is assumed, and all the explored points of the Potential Energy Surface contribute to the final spectrum. The calculation of the spectra directly includes environmental effects.

The use of ab initio/DFT MD guarantees the required accuracy in the description of both the forces between the atoms (governing the frequencies) and the electronic density fluctuations (governing the intensities). However, a method to efficiently predict the electric and magnetic dipole moment of the entire system at each time step of the MD is required. Moreover, for the condensed phase, a strategy to separate the contributions of the different components of a system (e.g., solute and solvent) is desired.

For instance, the total dipole moment can be obtained with the Berry phase approach or by the Wannier charges.^30,32,33^ In the case of the magnetic moment, a possible strategy^21^ is to use the nuclear velocity perturbation theory (NVPT) to compute the total magnetic moment. The spectrum is then posed in its molecular contributions in a second step, for instance, using localized molecular orbitals such as the maximally localized Wannier functions.^33,34^ While successfully predicting the VCD spectra, this method requires a substantial computational cost to compute the magnetic moment and localize the wave function. This load represents a massive obstacle to the standard application of this technique, especially when multiple scenarios must be tested to infer the correct one.

To reduce the computational cost, Thomas et al.^29^ proposed a semiclassical approach in which they reconstruct the magnetic response from the time-dependent electron density and assign the different molecular contributions by a Voronoi tessellation of the electron density. While this strategy strongly reduces the computational demands, the drawback is that it is based on a classical expression of the magnetic moment.

Here an alternative approach is proposed. This approach has been developed with two aims. The first aim is to bypass the cost of computing the total magnetic moment at each time step truly ab initio without introducing any classical approximation. The second aims is to partition the contributions to the spectrum in its various molecular components without any a-posteriori procedure for the localization of the wave function or the electronic density.

In the past, I have shown that it is possible to compute high-quality IR, Raman from the Fourier Transformation of the time-correlation functions of velocities, weighted by specific observables^35,36^ related to the activity of the vibrational modes. A similar Activity Weighted Velocities (AWV) correlation function strategy has also been proposed for the Sum Frequency Generation (SFG) spectra.^37−40^ These observables are the Atomic Polar Tensors (APT) for IR spectroscopy and the Raman tensors for Raman spectroscopy, and a combination of APTs and Raman tensors for SFG spectroscopy. This work adopts the same strategy to compute VCD spectra from DFT-MD simulations.

Section 2 illustrates the AWV method for the case of the VCD spectra. Section 3 critically assesses the implemented methodology to compute liquid phase spectra in the case of weak intermolecular interactions (section 3.1) and strong intermolecular interactions (section 3.2). Section 3.3 discusses the sensitivity of the method to anharmonic effects, large amplitude motions, and mechanical couplings. Section 4 draws some general conclusions and some guidelines.

## 2 Method

Within the time-correlation function formalism,^21,31^ the VCD absorption spectrum of an isotropic system can be computed from a classical nuclei trajectory as
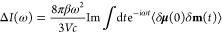
1where Im indicates the imaginary part of the integral, β = 1/*k*_b_*T*), *k*_b_ is the Boltzmann constant, *T* the temperature, ω is the vibrational frequency, ℏ = *h*/2π and *h* is Planck’s constant, *V* is the volume, **μ** and **m** are respectively the total dipole moment and the total magnetic moment of the system, δ **μ** (*t*) and δ **m** (*t*) their fluctuations with respect to their time average values.

In eq 1, a quantum correction factor, βℏ/(1 – exp(−βℏω)), has been applied to correct the classical line shape.^21,41^ For the sake of simplicity, the rest of the text will systematically use **μ**(*t*) instead of δ**μ**(*t*) and **m**(*t*) instead of δ**m**(*t*). However, the actual calculation still takes the fluctuations into account.

Thus, eq 1 can be rewritten as
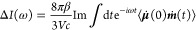
2where **μ̇** = d**μ**/d*t* and **ṁ** = d**m**/d*t*. The vectors **ξ**, **v**, and **a** are now introduced that collect, respectively, the 3N Cartesian coordinates of the N atoms of the system **ξ** = [*x*_1_, *y*_1_, *z*_1_, *x*_2_..., *z*_*N*_ ], the 3N Cartesian velocities **v** = [*v*_*x*__1_, *v*_*y*__1_, *v*_*z*__1_, *v*_*x*__2_..., *v*_*zN*_ ], and the 3N Cartesian accelerations **a** = [*a*_*x*__1_, *a*_*y*__1_, *a*_*z*__1_, *a*_*x*__2_..., *a*_*zN*_ ].

If there is no external field, **μ** is a function of the atomic position only, but not of the velocities. Therefore, **μ̇(***t*) can be expanded as
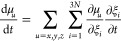
3where *μ*_*u*_ is the *u* = *x*,*y*,*z* component of total dipole moment **μ**. Recognizing that  is the *i*-th Cartesian velocity, whereas  is the *ui*-th element of the Atomic Polar Tensor **P**
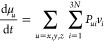
4

The magnetic moment,^42,43^**m**, contrary to **μ**, is a function of the atomic velocities, and for a closed-shell system . Therefore
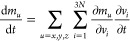
5where *m*_*u*_ is the *u* = *x*,*y*,*z* component of total magnetic moment **m**. By recognizing that  is the *i*-th Cartesian acceleration, whereas  is the *ui*-th element of the Atomic Axial Tensor **M**, one obtains
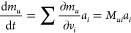
6Hence, eq 2 can be rewritten as
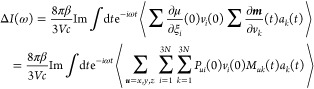
7where eq 7 has multiple computational advantages compared to eq 1. First, the time correlation function of **μ̇** and **ṁ** (and therefore **Pv** and **Ma**) decay faster^21^ than the one of **μ** and **m**. This allows the use of shorter simulations for the calculation of the correlation function. Second, the time evolution of **Pv** and **Ma** is less expensive to predicted than the one of **μ** and **m** or, **μ̇** and **ṁ**, as will be discussed in the next section.

Finally, velocities and accelerations are local quantities, and **P** and **M** elements are the first derivatives of the total dipole moment and *total* magnetic moment of the system with respect to a *specific* atomic coordinate/velocity. This opens the path for assigning the vibrational bands without any expensive wave function localization.^21,34^ In fact, the contribution of a fragment/molecule to the total signal can be obtained with no additional cost by separating the terms in eq 7
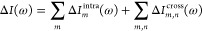
8where Δ*I*_*m*_^intra^(ω) is the contribution of molecule/fragment *m* to the total spectrum:
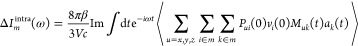
9and Δ*I*_*m,n*_^cross^ is the cross contribution that arises from the *m* and *n* fragments:
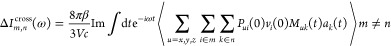
10

It is also possible to separate the contribution of the different conformers using a similar strategy as the one in ref (44) (see the Supporting Information for more details).

Notice that, contrary to methods based on the localization of wave function or electronic charge density, AWV uses physical observables, i.e., the APT and the AAT, for partitioning the signal into its molecular components.

### 2.1 Time Evolution of the Atomic Polar Tensor (APT) and the Atomic Axial Tensor (AAT)

Velocities and accelerations are readily available at each time step of the trajectory. Widespread QM codes, such as Gaussian,^22^ ADF,^23^ and Cp2k,^24^ allow the computing of APTs and AATs in the gas phase and periodic systems. However, evaluating them at each time step would be quite computationally expensive. While **μ**(*t*) changes on the same time scale of the atomic velocities (and therefore must be recomputed at each time step of the simulation), ref (35) shows that **P**(*t*) usually changes on a time scale that is 1–2 orders of magnitude slower than the atomic velocities. Therefore, **P**(*t*), at instant *t*, can be adequately described by a linear combination of the APTs of a set of appropriate reference structures:
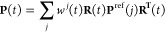
11where **P**^ref^ (*j*) is the APT of the *j*-th reference structure, *w*^*j*^ (*t*) is the probability that, at time *t*, the system is vibrating around the *j*-th reference structure, **R**(*t*) is the rotational matrix that guarantees the best overlap between **ξ** (ref) and the geometry in the MD simulation **ξ**(*t*), and **R**^**T**^(*t*) its transpose.

**R**(*t*) can be obtained by a quaternion fit that minimizes the sum of the squared distances between the mass weight coordinates of corresponding atoms.^45^ Such a rotation satisfies the Eckart conditions for small displacements^46^ and can still be used for large ones.^35^

To evaluate *w*^*j*^(*t*), a Gaussian distribution around the reference structures was assumed:^35,47^
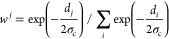
12where *d*_*j*_ is a metric measuring the “distance” between the geometry of the system at instant *t* and the reference structure *j*, and σ_c_ is the width of the Gaussian. The metric *d*_*j*_ must be chosen so that it is easy and cheap to be evaluated on the fly, but can still capture the fundamental differences between the reference structures. It has been shown^35,47^ that the following definition has the required characteristics:
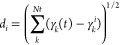
13where {*γ*_*k*_} can be a set of coordinates of the same type, e.g., all interatomic distances, all bond angles, all torsional angles, or all coordination numbers. Notice that in the case of the coordination number, the following continuous definition has been adopted:^48^
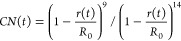
14where *r*(*t*) is the distance between the two atoms at time *t*, and *R*_0_ is a cutoff distance for the interactions.

If a set of coordinates of different types is required to univocally define the reference structures (for example, a combination of coordination numbers and torsional angles), the metric becomes a vector **d**_*j*_ = {*d*_*j*_^*x*^} and *w*^*j*^ can be generalized to^49^
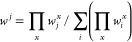
15with
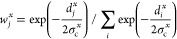
16where {*x*} is a subset of coordinates of the same type.

The strategy of eq 11 was successfully applied to obtain APTs, Raman tensors,^36^ and vibrational mode eigenvector matrix.^49^ Here, I proposed to extend the treatment to obtain AATs by a linear combination of the AATs of a set of appropriate reference structures:
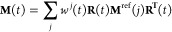
17

Notice that in the case of **M**, the gauge dependence needs to be correctly considered, i.e., the fact that, contrary to **P**, **M** is not translationally invariant. **M** can be computed in a distributed origin gauge^50,51^ (i.e., the derivatives are expressed in local coordinate systems with their origins on the moving atom). Then, the AAT in the laboratory reference system, M_*αk*_^Lab^, can be obtained from the one in the local reference system, M_*αk*_^Local^, by^52^
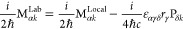
18where *ε*_*αγδ*_ is the antisymmetric unit third rank tensor (alternating tensor), *i* the imaginary unit, and *r*_*γ*_ is the relative position of the laboratory reference system with respect to the local one.

Finally, in the case of simulations under periodic boundary conditions, the “ill definition of the common origin” problem must be addressed. One possibility is to employ a similar formalism as the one proposed by Jahnigen et al.^53^ Another is to bypass the problem by averaging the predictions from multiple origins.^21,54^ This latter strategy will be adopted in section 3 via the average of the time correlation function of several independent trajectories. Because the case of disordered systems with fast enough dynamics will be discussed, this is enough to guarantee the gauge invariance of the computed spectra.

### 2.2 Divide and Conquer Strategy for APT and AAT Tensors

While eqs 8 and 17 strongly reduce the number of APTs and AATs that one needs to compute, at least all the explored minima of the PES must be included in the parametrization. When flexible structures and liquid phase systems are targeted, the number of required references increases almost exponentially with the degrees of freedom, imposing a substantial computational effort. This load can become a real bottleneck for using the method in the case of systems of hundreds of atoms, for which the computations of the APT and AAP tensors, even for a few reference structures, can be prohibitive.

However, it has been shown that APT and AAT tensors are usually transferable between fragments/clusters with the same local environment.^52,55−59^ The tensors can also be transferred between nonperiodic and periodic systems, with additional caution, in the case one is interested in absolute intensities, of taking into account the medium refractive index.^60^

Therefore, one possibility to bypass the problem is to use a multifragment strategy in which the system is divided in a set of N smaller relevant units. The APT, **P**_*i*_(*t*), and AAT, **M**_*i*_(*t*) of each unit *i* are then parametrized by independent calculations on model systems representative of the local environment of the unit/fragment.^35^
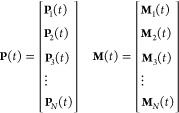
19

This approach allows dividing the original computational demanding problem into smaller ones. Local sets of reference structures can take the place of the shared global set. Additionally, for each unit/fragment, it is possible to substitute the global metric **d**_*j*_, with one specific only of the unit itself, **d**_***j***_^frag^.

Finally, to correctly describe the relative motion between units, it is possible to introduce a rotational matrix **R**_α∈frag_ that guarantees the best overlap between **ξ**_α∈frag_(ref) and the geometry in the MD simulation **ξ**_α∈frag_(*t*) of the {α} subset of atoms belonging to the unit.
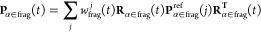
20
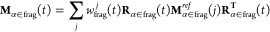
21Equations 19–21 allow transforming the scaling with the number of degrees of freedom from exponential to almost linear.

In the past, I have shown for IR and Raman spectroscopy that this divide and conquer strategy reduces the computational cost without losing accuracy.^35,36^ If proper sets of reference structures are chosen (see ref (35) for a discussion on how to select the reference structures), eq 20 (and its Raman respective) allows for correctly describing both large amplitude motions, such as the CH_3_ torsions, and changes of the intra- and intermolecular conformations. In the next section, it will be shown the same is true for VCD.

### 2.3 Chemically Equivalent Minima of the PES

Molecules/fragments often visit chemically equivalent minima of the potential energy surface, i.e., minima related to permutation of equivalent atoms, during a trajectory. One example of these equivalent minima is the three minima positions on the torsional potential energy surface of the CH_3_. Because the fragments/molecules are described by a Cartesian coordinates vector (**ξ**_frag_), the equivalent minima are distinct for the method described before. This must be taken into account. Indeed, it has been shown in the past that for obtaining high-quality IR spectra, it is essential to include all equivalent minima in the set of reference structures.^35^ One can expect the same for VCD. However, starting from a minimum, the tensors for an equivalent one can be easily obtained simply by permutating the proper elements (the ones of equivalent atoms) of the **P**_frag_^ref^ and **M**_frag_^ref^ tensors. Therefore, starting from the “*irreducible set*” (i.e., the set without the chemically equivalent minima), the “*reducible set*” (i.e., the complete set comprehensive also of the chemically equivalent minima) can be built without any additional computational cost.

### 2.4 Summary of the AWV Method

Our computational protocol for computing VCD spectra from DFT-MD simulations consists of the following steps:

1. Generation of a set of replicas of the system in the NVT ensemble
2. Running the DFT-MD trajectories in the NVE ensemble
3. Definition of the fragments in which to divide the system
4. Selection for each fragment an appropriate set of reference structures (able to adequately describe the intra- and supramolecular environment of the fragment)
5. Definition for each set of an appropriate metric *d*_*j*_ = {*d*_*j*_^*x*^}
6. Calculation of **P**^ref^ and **M**^ref^ tensors for the chosen “*irreducible*” set of reference structures
7. Generate the complete “reducible” set of **P**^ref^ and **M**^ref^ tensors, comprehensive also of chemically equivalent minima (see section 2.3 for more details)
8. Prediction of the time evolution of **P**(*t*) and **M**(*t*) of the system along the trajectory as the weighted sum of the **P**^ref^ and **M**^ref^ of relevant reference structures (eqs 19, 20, and 21)
9. Prediction of the time correlation function ⟨**P**(0) **v**(0) **M**(*t*) **a**(*t*)⟩ for each replica
10. Average the time correlation function of the different replicas
11. Compute the VCD spectrum by eq 7
12. Assign the VCD spectrum by eqs 9 and 10

## 3 Benchmark

**Figure 1:**
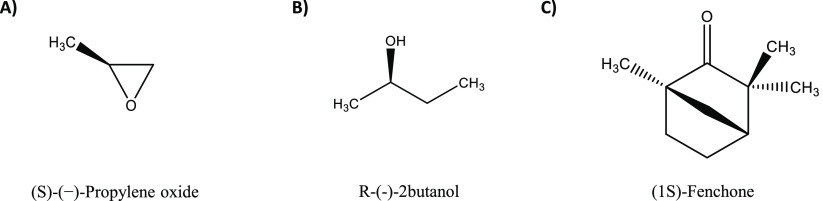
Molecular structure of the (*S*)-(−)-Propylene oxide (S-PO), the (*R*)-(−)-2-butanol (R2B), and of the (1*S*)-Fenchone (1S-FEN).

To critically rate the performance of the AWV method, the algorithms will first be tested in the case of a rigid molecule in the bulk phase when only weak intermolecular interactions are present (section 3.1). For this, (*S*)-(−)-Propylene oxide in the liquid phase will be used (Figure 1A).

Then, the AWV method will be assessed when strong intermolecular interactions are present and multiple conformations contribute to the spectrum (section 3.2). For this, (*R*)-(−)-2-butanol in neat liquid phase will be used (Figure 1B).

Finally, the strength and weakeness of DFT-MD simulations coupled with the AWV in describing anharmonicity and mechanical couplings effects on the spectra will be evaluated (section 3.3). This will be done anlizing the spectrum of (1*S*)-Fenchone molecule in the gas phase (Figure 1C).

### 3.1 Weakly Interacting Systems: (*S*)-(−)-Propylene Oxide in the Liquid Phase

As a first benchmark for the AWV method, the liquid-phase VCD spectrum of the (*S*)-(−)-Propylene oxide (S-PO) molecule is analyzed. The S-PO spectrum, both in the gas and liquid phase, has been extensively studied in the literature^61−64^ because it is one of the smallest chiral molecules, making this a perfect test case for the proposed method.

Due to the weak intermolecular interactions between the S-PO molecules, it is expected that the main features of the liquid-phase experimental spectrum in the fingerprint region are already well reproduced by the gas-phase calculations. Therefore, a comparison with gas-phase spectra in the double harmonic approximation will be also discussed.

#### 3.1.1 Computational Details

The *static* spectra on the gas-phase S-PO molecule discussed in the next section have been computed with the Gaussian16 code,^22^ VCD package,^65,66^ in the double harmonic approximation. The B3LYP functional,^67,68^ augmented with the Grimme D3 dispersion term,^69^ and the aug-cc-TZvp basis set^70,71^ have been chosen.

The *dynamic* spectra discussed in section 3.1.2 have been computed using eq 7. The DFT-MD trajectories on the liquid phase of the S-PO molecule have been run with the Cp2k code.^24^ Born–Oppenheimer molecular dynamics were used, i.e., at each time step, the electronic wave function was converged, imposing a threshold for the energy difference between two SCF cycles of 3.0 × 10^–7^ Hartree/cell. The classical Newton equations of motion for the nuclei were integrated through the velocity Verlet algorithm with a time step of 0.4 fs.

Static harmonic calculations demonstrate that for this molecule, the gas-phase spectra predicted by the BLYP-D3 functional show qualitative agreement with the more expensive B3LYP-D3 ones, with the main difference between the two being a general redshift of the bands in the BLYP spectrum compared to the B3LYP (see section S2 of the Supporting Information for more details). Therefore, the BLYP functional,^67,72^ augmented with the Grimme D3 dispersion term,^69^ has been preferred for the DFT-MD simulations as a reasonable compromise of accuracy and computational cost. A hybrid Gaussian and plane waves (GPW) basis set, consisting of a 400 Ry energy cutoff plane-wave basis set, coupled with the TZVP-MOLOPT-GTH basis set, was selected. Pseudopotentials of the GTH type (Goedecker-Teter-Hutter)^73^ were also adopted. The simulation consisted of 16 molecules in a cubic box of 12.296 × 12.296 × 12.296 Å. The box sizes were chosen to reproduce the experimental density at room temperature (0.83 g/cm^3^), and periodic boundary conditions were applied in all three spatial directions to mimic a bulk liquid phase.

Three independent replicas of the system were generated by extracting sets of atomic positions from a classical MD trajectory, each separated from the others by one nanosecond at least. These sets were used as a starting point for the DFT-MD simulations. The following computational protocol were adopted. For each replica, an NVT trajectory of 5 ps was run to equilibrate the system at 300 K. A CSVR thermostat^74^ (time constant 300 fs) was applied together with the automatic rescaling of the velocities each time the temperature fluctuations exceed the threshold of 300 K ± 30 K. Subsequently, the thermostat is turned off, and a trajectory of 20 ps is produced in the NVE ensemble. Once **ξ**(*t*) and **v**(*t*) are obtained, the accelerations **a**(*t*) can be computed by numerical differentiation. A five-point central difference formula (see section S1 of the Supporting Information for details) was chosen to guarantee a negligible numerical error. From **v**(*t*) and **a**(*t*), the ⟨**P**(0) **v**(0) **M**(*t*) **a**(*t*)⟩ correlation function was computed for each replica. The time evolutions of **P** and **M** were predicted by eqs 20 and 21.

Four fragments model each molecule: the CH_3_ group, the hydrogens of the CH_2_, the hydrogen of the CH, and the C–O–C ring. Because of the weak interactions between the molecules in the bulk phase, it is reasonable to consider that the effect of the intermolecular environment on the tensors on each solvated molecule is small. Therefore, for all four fragments, the required reference structures consist of a single gas S-PO molecule in the different positions along the CH_3_ rotational Potential Energy Surface. In particular, a *reducible* set of 3 reference structures, i.e., only one structure in the *irreducible* set, shows to be enough (see section S3 of the Supporting Information for more details).

Notice that while the reference structures consist of a whole S-PO molecule, each molecule still needs to be modeled by multiple fragments to be able to describe the possible large amplitude motions, especially of the CH_3_ groups, as demonstrated in ref.^35^ in the case of the IR spectra.

The **P**^ref^ and **M**^ref^ tensors of the set of reference structures have been evaluated with the same computational setup as the *static* spectra (Gaussian16 code, B3LYP-D3/aug-cc-TZvp).

Finally, the correlation function used in eq 7 is obtained as the average of the correlation functions computed for each replica.

#### 3.1.2 Results and Discussion

**Figure 2:**
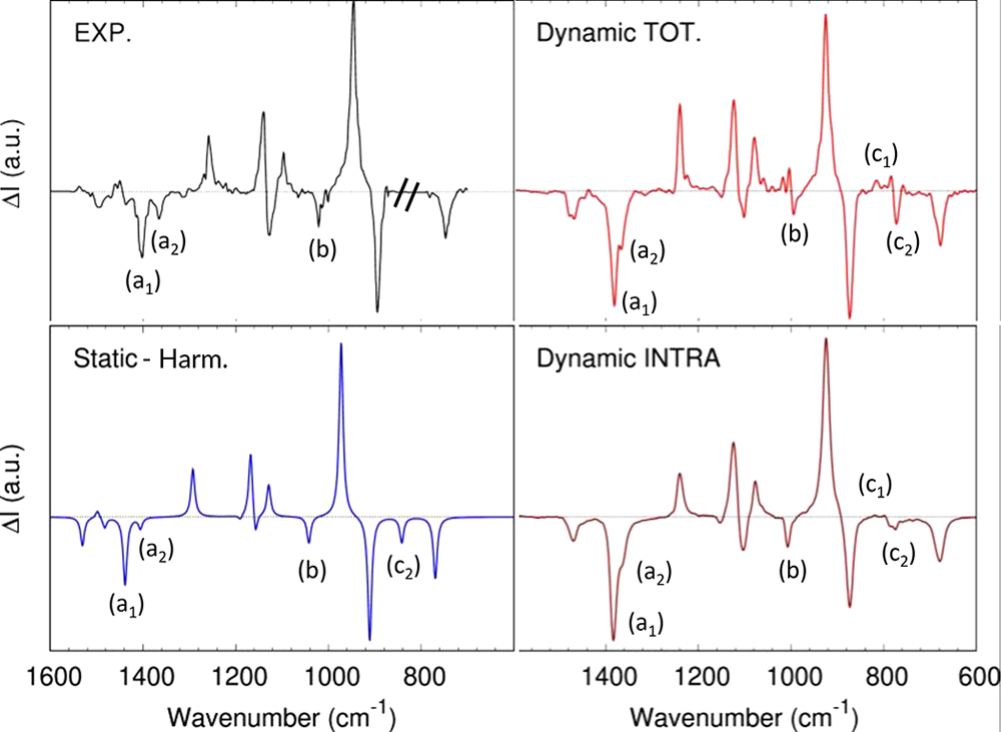
(*S*)-(−)-Propylene oxide (S-PO) VCD spectrum. Top left: experimental neat liquid-phase spectrum, reproduced from ref (64). Bottom left: computed gas-phase *static* spectrum on the isolated molecule in the double harmonic approximation. Top right: total *dynamic* liquid-phase spectrum computed with the AWV method (eq 7). Bottom left: intramolecular component of the *dynamic* liquid-phase spectrum computed with the AWV method (eq 9).

Figure 2 shows the experimental spectrum of the neat liquid of S-PO,^64^ the gas-phase *static* spectrum predicted in double harmonic approximation, and the liquid-phase total and intramolecular *dynamic* spectra computed with the AWV method (respectively eqs 7 and 9).

Because of the weak intermolecular interactions and the existence of only one possible conformer for the S-PO molecule, it is expected that the *static* spectrum already provides a good match with the experiments. In fact, the *static* spectrum reproduces well all the main features of the experimental spectrum. However, even in this simple case, the *dynamic* spectrum provides some improvements compared to the harmonic *static* one. For example, the *dynamic* spectrum better describes the complexity of the 1350–1450 cm^–1^ spectral region than the *static* spectrum. Where the experimental spectrum points to a set of convoluted peaks, the *static* spectrum predicts only two narrow bands for this frequency region: the 1400 cm^–1^ band (Figure 2, a_1_) belonging to a combination of the CH and CH_3_ bending, and the 1362 cm^–1^ band (Figure 2, a_2_), mostly coming from the CH_3_ umbrella motion.

Instead, the *dynamic* spectrum correctly predicts a set of broader features. In the past, anharmonic calculations^61^ showed that many overtones and combinations bands are active in the frequency range between 1300 and 1500 cm^–1^. The *static* harmonic calculations cannot predict them, while *dynamic* simulations can at least partially describe them, which explains the better match with the experiments. Still, the classical nuclei movement does not allow a perfect description of these phenomena; therefore, some mismatches are still present. The performance of AWV will be discussed in describing large amplitude anharmonic motion and combinations bands more quantitative in section 3.3.

In any case, for S-PO, there are only a few exceptions to this equal or better performance of the *dynamic* spectrum compared to the *static* one. The two bands at 1362 and 1400 cm^–1^ (Figure 2, a1 and a2) of the experimental spectrum are merged in a single broad feature in the *dynamic* spectrum, whereas they are correctly split in the *static* spectrum. However, this is partly due to the choice of the BLYP functional for the *dynamic* instead of the more accurate B3LYP used for the *static* spectrum. Static calculations on the gas-phase molecule show a frequency shift between the two bands of 34 cm^–1^ at the B3LYP level (similar to the 38 cm^–1^ experimental one) compared to the 20 cm^–1^ predicted at the BLYP level (see section S2 of the Supporting Information).

Another interesting case is the 1023 cm^–1^ band (b), associated in the past with one of the two degeneracy-lifted methyl rocking^63^ features (the other is the strong positive feature at 950 cm^–1^). The *static* spectrum correctly predicts a negative intensity for this band, and the normal-mode analysis of the gas-phase spectrum shows that there is only one normal mode in this frequency range, and it is a combination of CH_2_ twisting, CH wagging, and CH_3_ rocking. In the total *dynamic* spectrum is seen a broad band with multiple components overlapping in which a negative band follows the first set of positive peaks. The experimental spectrum in this region shows a broad feature possibly underlining multiple components, as predicted by the *dynamic* spectrum, but no relevant positive bands. Interestingly, decomposing the *dynamic* signal in its intramolecular and intermolecular components (eq 9), it can be seen that the overlap of an intermolecular (positive) component to the (negative) intramolecular one generates this feature (Figure 2 panel top-right and bottom right). The mismatch of the *dynamic* spectrum compared to the experimental one can possibly be ascribed to the chosen limited simulation box size that, being not too large, might artificially induce a supramolecular organization. Another possible player is the BLYP level of theory. It cannot be excluded that a better functional would give the correct description.

Interestingly, in the region below 900 cm^–1^, another set of features (Figure 2, c1 and c2) is quite sensitive to the intermolecular environment. The total *dynamic* spectrum shows a set of positive bands (c1) followed by a negative one (c2), while if the intramolecular component is looked at, only the negative band last. The *static* gas-phase spectrum also shows only a negative band that rises from a combination of the CH_2_ wagging, CH wagging, CH_3_ rocking, and C–O stretching. Therefore, the ratio between c1 and c2 could be a marker of the intermolecular interactions. Unfortunately, the region between 880 and 800 cm^–1^ is challenging to be measured from the experimental point of view since the corresponding IR band (829 cm^–1^) is exceptionally intense. In ref, (64) this region is not reported. Another set of experiments (ref (63)) shows two small positive bands around 859 and 875 cm^–1^ (see section S4 of the Supporting Information). However, the intensity of the two peaks is almost of the same magnitude as the signal noise. Therefore, conclusions cannot be made on these bands and thus we will not comment any further.

### 3.2 Strongly Interacting Systems: (*R*)-(−)-2-Butanol in the Liquid Phase

**Figure 3:**
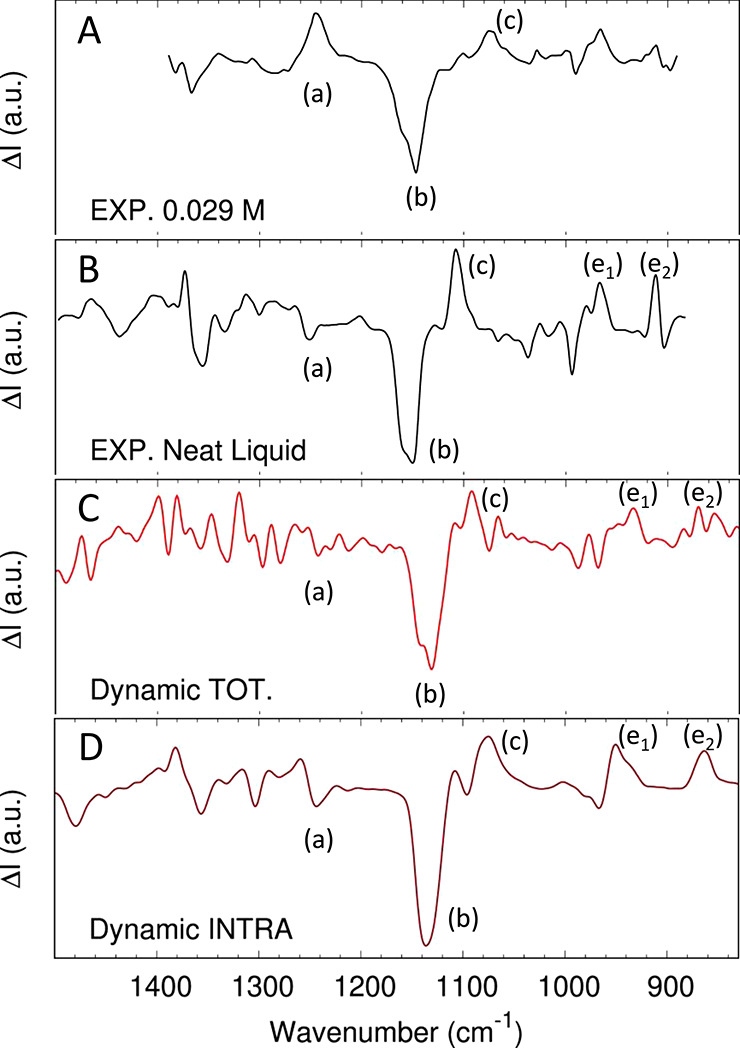
(*R*)-(−)-2-butanol (R2B) in liquid-phase VCD spectrum in the fingerprint frequency region. Panel A: diluted experimental spectrum, reproduced from ref (75) (0.029M/CS_2_ solution). Panel B: neat liquid experimental spectrum, reproduced from ref (75). Panel C: total *dynamic* liquid-phase spectrum computed with the AWV method (eq 7). Panel D: intramolecular component of the *dynamic* liquid-phase spectrum computed with the AWV method (eq 9).

As a second benchmark for the AWV method, the liquid-phase VCD spectrum of the R2B molecule is analyzed. R2B has also been studied in the past,^27,29,75^ and the reader is referred to these previous works for the assignment of the vibrational modes. The R2B molecule is characterized at room temperature by nine stable intramolecular conformers.^27,28^ On top of this, strong hydrogen bonds are formed in the liquid phase. These have a non-negligible effect on the spectrum; consequently, the single isolated molecule is not a sound model system in this case. Notice in fact that, when compared to the spectrum of high diluted solution (Figure 3), the experimental spectrum of the neat liquid R2B shows the disappearance^75,76^ of the 1241 cm^–1^ band (a) and the redshift of the 1176 cm^–1^ positive peak (c) to 1107 cm^–1^. Both peaks are related to the COH bending motions; therefore, not surprisingly, they are sensitive to the intermolecular environment.

Also, monomers immersed in an implicit solvent cannot model the effect of the hydrogen bonds on the spectrum.^19,28^ The hydrogen bonds must be explicitly considered in the calculations. This implies the need for a correct sampling of both the intra- and intermolecular conformations, increasing exponentially the dimensions of the conformational space that must be mapped. This requirement makes the prediction of the liquid-phase spectra of systems such as R2B with *static* calculations not trivial, as already discussed in the introduction of this paper. In particular, it is exceptionally challenging when limited structure variations, such as an intermolecular hydrogen bond vibration, determine significant modulations of the band shapes.^19^

Instead, it is quite easy to predict the spectra of liquid-phase systems with either weak or strong intermolecular interactions, with *dynamic* calculations using eq 1 or eq 7. In the following sections, it will be seen how in particular, AWV *dynamic* calculations allow obtaining straightforward good spectra at a relatively reduced computational cost.

#### 3.2.1 Computational Details

The *dynamic* spectra, discussed in section 3.2.2, have been computed using eq 7. The DFT-MD trajectories on the liquid phase of the R2M molecule were run with the Cp2k code.^24^ Born–Oppenheimer MDs were used, i.e., at each time step, the electronic wave function was converged, imposing a threshold for the energy difference between two SCF cycles of 3.0 × 10^–7^ Hartree/cell. The classical Newton equations of motion for the nuclei were integrated through the velocity Verlet algorithm with a time step of 0.4 fs.

The simulation consists of 32 molecules in a cubic box of 16.956 × 16.956 × 16.956 Å. The box sizes were chosen to reproduce the experimental density at room temperature (0.808 g/cm^3^), and periodic boundary conditions were applied in all three spatial directions to mimic a bulk liquid phase.

Because of the dimension of the simulated systems, the BLYP functional,^67,72^ with the D3 Grimme correction for dispersion,^69^ was chosen. A hybrid Gaussian and plane waves (GPW) basis set, consisting of 400 Ry energy cutoff plane-wave basis set, coupled with the set DZVP-MOLOPT-GTH-SR basis set pseudopotentials of the GTH type (Goedecker-Teter-Hutter)^73^ were also adopted. This computational setup is a good compromise between accuracy and computational cost, as already shown by other authors.^27,29^

Eight independent replicas of the system were generated by extracting a set of atomic positions from a classical MD trajectory each nanosecond. These sets were used as a starting point for the DFT-MD simulations. The following computational protocol was adopted. For each replica, an NVT trajectory of 5 ps was run to equilibrate the system at 300 K. A CSVR thermostat^74^ (time constant 300 fs) was applied together with the automatic rescaling of the velocities each time the temperature fluctuations exceeded the threshold of 300 *K* ± 30 *K*. Subsequently, the thermostat was turned off, and a trajectory of 20 ps was produced in the NVE ensemble. Once **ξ**(*t*) and **v**(*t*) were obtained, the accelerations **a**(*t*) could be computed by numerical differentiation. A five-point central difference formula (see section S1 of the Supporting Information for details) was used to guarantee a negligible numerical error. From **v**(*t*) and **a**(*t*), the ⟨**P**(0) **v**(0) **M**(*t*) **a**(*t*)⟩ correlation function was computed for each replica. Finally, the correlation function used in eq 7 was obtained as the average of the correlation functions computed for each replica.

The time evolution of **P** and **M** were predicted by eqs 20 and 21. Following the divide and conquer strategy detailed in section 2.2, each molecule in the system was modeled by five fragments: the OH group, the two CH_3_, the CH_2_, and the central CH. For each of the fragment a different local metric **d**_***j***_^frag^ was defined (see section S5 of the Supporting Information for more details).

The following protocol was used to define the set of reference structures. The eight DFT-MD trajectories were analyzed molecule by molecule, focusing on the intramolecular degrees of freedom. The explored molecular conformations were classified in terms of intramolecular torsional angles. Subsequently, 107 not-equivalent conformations (that become 963 in the *reducible* set) were selected (see section S5 of the Supporting Information for the tests with other sets). For each conformation, a cluster composed of a target R2M molecule with the right torsional angles and all the other R2M molecules H-bonded to it was extracted from the DFT-MD trajectories. The APTs and the AATs of the set of reference structures have been computed on these model systems with single points calculations with the Gaussian16 code,^22^ VCD package.^65,66^ The B3LYP functional and the 6-331++G** basis set^70,77^ have been shown in the past^35^ to be a reasonable compromise between accuracy and computational cost to reproduce the vibrational intensities for this kind of system. Therefore, it was also adopted here.

#### 3.2.2 Results and Discussion

In Figure 3, the total (eq 7) and intramolecular *dynamic* (eq 9) spectra computed with the AWV method for the liquid phase of the R2B molecule are compared to the experimental spectra for the neat liquid phase and a high diluted solution of R2B (0.029M/CS_2_ solution). The predicted liquid-phase spectrum for the R2B molecule (Figure 3, panels C and D) are red-shifted compared to the experimental one (panel B). However, this is related to the choice of the BLYP functional, as previous studies have pointed out.^27,29^ Apart from this, the AWV method reproduces all the main features of the experimental spectrum of the liquid phase quite well: a crowded region between 1400 and 1200 cm^–1^, a strong negative band around 1155 cm^–1^ (b), followed by a positive peak at 1105 cm^–1^ (c), a negative band around 993 cm^–1^(d), and two positive ones at 967 and 908 cm^–1^ (e1 and e2). The previously recognized diagnostic markers of the liquid phase, i.e., the absence of the positive peak at 1241 cm^–1^ (a) and the appearance of a strong narrow peak at 1107 cm^–1^ (c), are correctly mimicked by the AWV method. The computed total *dynamic* spectrum quality between 800 and 1200 cm^–1^ is high. For example, AWV can correctly predict the splitting of the strong negative feature around 1155 cm^–1^ (b) related to the CCH bending vibration. Instead, between 1200 and 1400 cm^–1^, the comparison with the experimental spectrum is slightly less good. In particular, the computed spectrum shows too narrow bands compared to the experimental ones. This can be partially related to the computational setup, chosen as a compromise between accuracy and computational cost, that cannot completely mimic the heterogeneity of the real liquid. One possible solution to further refine the description of this spectral region is to increase the number of independent replicas used to compute the spectrum; another is to use a larger simulation box. In any case, the current simulation setup allows us to clearly predict the main features of the R2B experimental spectrum. Therefore, the result is accurate enough for the purpose of this paper, and these effects will not be further investigated.

To conclude the discussion on this benchmark system, the focus now moves to comparing the computed total *dynamic* spectrum to its intramolecular component (Figure 3, panels C and D). On the one hand, the intramolecular component converges with fewer trajectories than the total spectrum (only two instead of eight, see section S6 of the Supporting Information details). This latter requires an extended sampling due to the cross-correlations component. One interesting point, therefore, is to understand to which extent the cheaper intramolecular component can be reasonably used to assign the experimental spectra as a substitute for the more expensive total spectrum when large (i.e., computationally expensive) systems are under study.

In the case of the R2B molecule, for the fingerprint region, the intramolecular liquid-phase spectrum misses some features compared to the total one, but the main bands of the experimental spectrum (b,c,d,e1,e2) are already there. Therefore, for this case, the intramolecular component (eq 9) of the total signal (eq 7) can be a fast alternative for a qualitative spectrum assignment.

The generalization of this conclusion to other molecules and spectral ranges must be made cautiously. Compared to the total spectrum, the intramolecular component can correctly describe local vibrations taking into account the effect of the liquid-phase environment (the MD simulations are still in the liquid phase). However, it fails in the case of collective motions for which the intermolecular cross-correlation contributions strongly modulate the signal.^78^ Indeed, for S-PO (section 3.1.2), the comparison of the total spectrum and intramolecular component has been used to quantify the degree of delocalization of each mode.

### 3.3 Anharmonic Effects: the (1*S*)-Fenchone Gas-Phase Spectrum

As a final benchmark for the AWV method, the gas-phase VCD spectrum of (1*S*)-Fenchone (1S-FEN) was analyzed. This spectrum has also been extensively studied in the literature.^79,80^ In particular, see the detailed assignment of the vibrational bands by DFT calculations done by Longhi et al.^79^ 1S-FEN is interesting as a test case since the presence of multiple CH_3_ groups able of large amplitude motions.

In section 3.3.3, the *static* spectrum, computed in double harmonic approximation, will be compared with the *dynamic* ones obtained with eq 7 for both the fingerprint region and the CH stretching region. In the case of the *dynamic* spectra, the temperature will be played with as a means to explore different portions of the Potential Energy Surface. Since the 1S-FEN has only one possible conformer, increasing the temperature, the strength and limitations of the proposed method can be more critically assessed when anharmonic effects and mechanical couplings start to play a role.

#### 3.3.1 Computational Details

The *static* spectrum of the gas-phase isolated 1S-FEN molecule discussed in the next section was computed with the Gaussian16 code,^22^ VCD package,^65,66^ in the double harmonic approximation. Previous works^20^ have shown that the B3LYP functional^67,68^ with the aug-cc-TZvp bais set^70,71^ allows for correctly reproducing all the main features of the experimental spectrum in the fingerprint region. Therefore, the same computational setup was adopted here.

The *dynamic* spectra discussed in section 3.3.3 was computed using eq 7. The DFT-MD trajectories on the gas phase of 1S-FEN molecule were run with the Cp2k code.^24^ Born–Oppenheimer MDs were used, i.e., at each time step, the electronic wave function was converged, imposing a threshold for the energy difference between two SCF cycles of 3.0 × 10^–7^ Hartree/cell. The classical Newton equations of motion for the nuclei were integrated through the velocity Verlet algorithm with a time step of 0.4 fs. Because the small dimensions of the molecule allow it, the B3LYP functional was also used for the DFT-MD simulations in this case. A hybrid Gaussian and plane waves (GPW) basis set, consisting of 400 Ry energy cutoff plane-wave basis set, coupled with the TZVP-MOLOPT-GTH basis set, was selected but for the Fock exchange. Due to the enormous computational cost of this letter term, the auxiliary cpFIT3 basis set was employed instead with the Auxiliary Density Matrix Methods (ADMM).^81^ Pseudopotentials of the GTH type (Goedecker–Teter–Hutter)^73^ were also adopted.

The 1S-FEM molecule has been simulated in a periodic box 12.0 × 12.0 × 12.0 Å that has shown to guarantee negligible interactions with the periodic replica.

The spectra have been simulated at three different temperatures (50, 300, and 600 K) to critically assess the effect of the progressive inclusion of anharmonic effects. The following computational protocol was adopted. An NVT trajectory is run for each temperature to equilibrate the system at the target temperature. A CSVR thermostat^74^ (time constant 300 fs) has been applied together with the automatic rescaling of the velocities each time the temperature fluctuations exceed the threshold of ±30*K*.

After the first 10 ps were deleted, an ensemble of independent replicas for the system was created from this trajectory by extracting each 5 ps a set position, **ξ**(0), and velocities, **v**(0), for the atoms of the system. These sets were used as a starting point of a production run (of 20 ps) in the NVE ensemble. Once **ξ**(*t*) positions trajectory was obtained, to remove possible artificial contributions from the rotations, a rotation-free trajectory was computed by
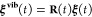
22where **R**(*t*) is the rotational matrix that guarantees the best overlap between **ξ**(*t*) and the initial geometry **ξ**(0). **R**(*t*) can be again obtained by a quaternion fit that minimizes the sum of the squared distances between the mass weight coordinates of corresponding atoms.^45^ Once **ξ**^vib^(*t*) was computed, the velocities **v**^vib^(*t*) and the acceleration **a**^vib^(*t*) were calulated by numerical differentiation. A five-point central difference formula was used (see section S1 of the Supporting Information for details) to guarantee a negligible numerical error on the kinetic energy.

From **v**^vib^(*t*) and **a**^vib^(*t*), the ⟨**P**(0) **v**^vib^(*t*) **M**(*t*) **a**^vib^(*t*)⟩ correlation function was computed for each replica. The time evolution of **P** and **M** tensors were predicted by eqs 20 and 21. The **P**^ref^ and **M**^ref^ tensors of the set of reference structures were evaluated with the same computational setup as the *static* spectra (Gaussian16 code, B3LYPD3/aug-cc-TZvp). Four fragments modeled the system to describe the possible CH_3_ large amplitude motions: the three CH_3_ groups and the central body of the molecule (see section S7 of the Supporting Information for more details). Because of the small dimension of the system, for all four fragments, the required reference structures consisted of complete 1S-FEM molecules.

Finally, the correlation function used in eq 7 was obtained as the average of the correlation functions computed for each replica. Three trajectories were enough at 50 K to ensure a well-converged spectrum, while 8 and 14 were required at 300 and 600 K (see section S8 of the Supporting Information for more details). This is due to the activation, at the temperature increase, of large, anharmonic amplitude motions of the CH_3_ groups, and their mechanical couplings to other modes that require additional sampling.

#### 3.3.3 Results and Discussion

**Figure 4:**
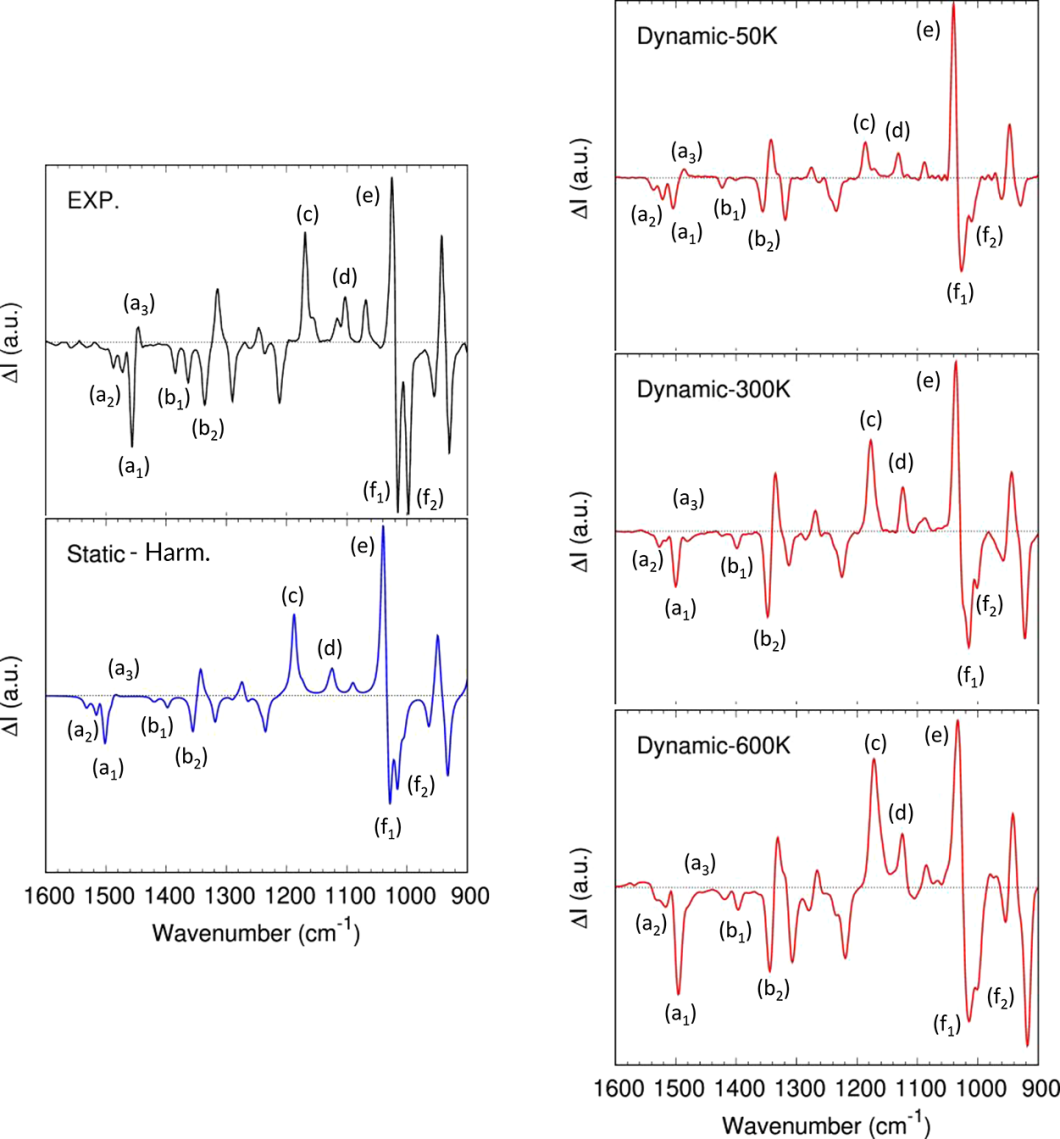
(1*S*)-Fenchone (1S-FEN) VCD spectrum in the fingerprint frequency region. Top left: experimental spectrum, reproduced from ref (79) (1.2 M/CCl_4_ solution). Bottom left: computed gas-phase *static* spectrum on the isolated molecule in the double harmonic approximation. Right: *dynamic* gas-phase spectra of the isolated molecule computed with the AWV method (eq 7) at 50, 300, and 600 K. All the spectra were renormalized on the (e) band (1023 cm^–1^ in the experimental spectrum).

Figure 4 shows the experimental spectrum of 1S-FEN^79^ (1.2 M/CCl_4_ solution), the gas-phase *static* spectrum predicted in double harmonic approximation, and the gas-phase *dynamic* spectra computed with the AWV method (eqs 7 and 9) at 50, 300, and 600 K. All the spectra are normalized on the 1023 cm^–1^ experimental band (Figure 4e).

First, consider the results of the MD simulations at 50 K. As expected, the *dynamic*-50K spectrum is similar to the *static* harmonic one. Due to the low temperature, the system can explore only the bottom of the potential energy well. No change of conformation occurs, no large amplitude motions are activated, and therefore most of the vibrations are expected to be harmonic. If the details are reviewed, both methods correctly predict the three negative bands between 1450 and 1486 cm^–1^ in the experimental spectrum (Figure 4, a1 and a2). However, both the *static* and the *dynamic*-50K underestimate the intensity of the experimental feature at 1455 cm^–1^ (a1). Instead, if the tiny band around 1446 cm^–1^ (a3) is considered, the *dynamic*-50K spectrum gives a slightly better description of the intensity of this peak. Actually, the *static* harmonic approximation also predicts the band but fails to give the right intensity to this band.

Moving on to lower frequencies, it can be seen that the two negative bands at 1384 and 1363 cm^–1^ (b1) are predicted but underestimated in intensity compared to the 1335 cm^–1^ band (b2) by both the *static* and the *dynamic*-50K spectra. The band at 1168 cm^–1^ (c) is well predicted in the *static* spectrum but underestimated by the *dynamic*-50K one. Consequently, the *dynamic*-50K overestimates the intensity ratio between the 1023 cm^–1^ band (e) and the 1168 cm^–1^ band (c). The splitting of the two experimental bands at 1105 and 1115 cm^–1^ (d) is underestimated by the *static* and the *dynamic*-50K calculations generating a single broad feature. Finally, the ratio between the two intense negative bands at 1015 and 995 cm^–1^ (f1, f2) is inverted by both *static* and *dynamic*-50K simulations, but somehow more by the latter.

**Figure 5:**
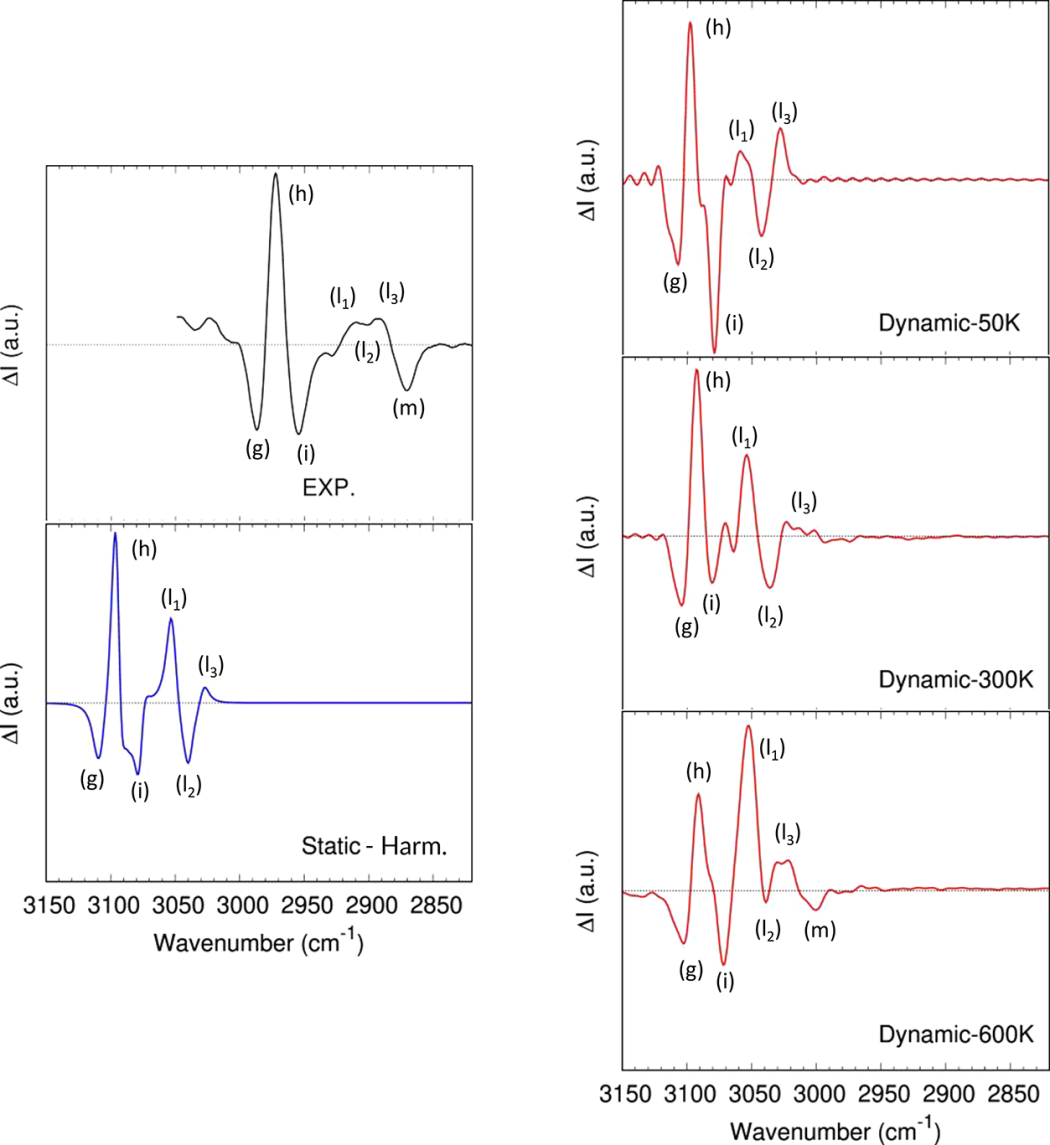
(1*S*)-Fenchone (1S-FEN) VCD spectrum in the CH-stretching frequency region. Top left: experimental spectrum, reproduced from ref (79) (0.3 M/CCl4 solution). Bottom left: computed gas-phase *static* spectrum on the isolated molecule in the double harmonic approximation. Right: *dynamic* gas-phase spectra of the isolated molecule computed with the AWV method (eq 7) at 50, 300, and 600 K.

Even with these differences, the global comparison with the experiments is good for both the *static* and *dynamic*-50K methods in the fingerprint region. Looking instead at the high-frequency range (Figure 5), the comparison with the experiment is less good. Both calculations show the well-known strong blue shift of the complete set of active bands due to the missing description of the anharmonicity. Moreover, while it can still be recognized the main features related to the CH_3_ and CH_2_ antisymmetric vibrations (Figure 5g the negative band around 2986 cm^–1^, (h) the positive band around 2972 cm^–1^, and (i) the negative band around 2953 cm^–1^), things become more uncertain in the region of the symmetric stretching and in particular the negative band below 2900 cm^–1^ (m) in the experiment is completely missing in the computed spectra. This band has been attributed^79,80^ to a Fermi resonance of the symmetric CH_2_ stretching. Therefore, it is not surprising that it is not described by the *static* calculations in double harmonic approximation but also by a classical MD simulation in which the system explores only the (harmonic) bottom of the potential well.

Next, consider the spectra obtained at 300 and 600 K. These spectra must be examined with some caution. While the position and the sign of the bands are statistically stable, the relative intensities of the bands are not still well converged with 14 independent trajectories (see section S8 of the Supporting Information). This can be related to the fact that this part of the spectrum is quite sensitive to the anharmonicity, both in terms anharmonic shape of the Potential Energy Surface of the modes and the activation of the coupling with lower frequencies modes. Extensive sampling is needed to converge the data.

With this in mind, some observations can be made. The vibrational bands redshift at increasing temperatures due to a significant exploration of the anharmonic portion of the PES, providing a better match with the experiments. However, the computed frequencies are still far from the experimental ones because of being much below the Zero Point Vibrational Energy of a CH stretching (these would require simulation at more than 4000 K). Another interesting aspect of the high-temperature simulations is that the symmetric stretching region (Figure 5, l1, l2, l3, m bands) substantially evolves with the increase of the temperature (i.e., at the inclusion of the anharmonic effects). In contrast, the effect on the asymmetric stretching bands (Figure 5, g, i, h bands) is more limited. This points to a stronger mechanical coupling between the lower frequency vibrations and the symmetric stretching modes compared to the antisymmetric ones. At last, it can be noticed that at 600 K, the negative band below 2900 cm^–1^ starts to be visible.

Let us now move back to the lower frequency region. This spectral region seems to be less sensitive to sampling issues, even at 600 K (see Supporting Information, section S8). Therefore, the discussion can be more quantitative. Below 1600 cm^–1^, the description of the spectrum (that, in any case, was already good at 50 K) is generally improved going to a higher temperature. One example is the already discussed set of bands around 1450 cm^–1^ (Figure 4, a1 and a2). These bands are a convolution of a set of positive and negative bands, and they can be assigned to the difference in-phase and out-of-phase combinations of HCH bending of the CH_2_ and CH_3_. At 600 K, the intensity ratio between panels a1 and a2 is finally correctly predicted. Also, the intensity ratio of the 995–1015 cm^–1^ doublet, coming from a combination of CH_2_, CH_3_ twisting, and rocking motions + (O)C–C stretching, is enormously improved. In the case of the negative bands at 1384 and 1363 cm^–1^ (b1), generated by in-phase and out-of-phase combinations of CH_3_ umbrella motions, the *dynamic-*300 K is enough to predict these features correctly. Notice how all the discussed bands have the CH_3_ vibrational modes in common. Considering the nature of these vibrational modes, the improvement in the *dynamic* spectra can be related to a better description of the CH_3_ and the activation of mechanical coupling with the large amplitude CH_3_ torsions.

There are a few exceptions to this general improvement of the spectra. One example is the positive bands at 1446 cm^–1^, better described at 50 K. Possibly, our level of theory does not entirely well describe the mechanical coupling with the large amplitude CH_3_ torsions for this band. Another is the 1168 cm^–1^ band that is gaining too much intensity as the simulation temperature increases.

In general, it can be concluded that the AWV method is quite sensitive to the anharmonic shape of the potential energy surface and mechanical coupling between the modes. If the right energy is assigned to the system (the modes under analysis are not too far too their ZPVE), the method has the means to describe these effects.

## 4 General Discussion and Conclusions

The AWV method presented here is suitable for predicting the VCD spectra in the fingerprint region of systems in the gas phase and liquid phase. The match with the experimental spectra for the three presented benchmark systems is striking.

Compared to *static* calculations, AWV provides an easy way to predict the anharmonic spectra of disordered systems with strong intermolecular interactions. For example, AWV allows one to efficiently handle also situations in which significant modulation of the band shapes is determined by limited structure variations, without any advanced clustering technique and/or ad-hoc models. However, notice that in the case of gas-phase and/or weakly interacting condensed phase systems with a strong harmonic character, the computational cost of AWV is still much higher than the standard *static* double harmonic calculations, and the latter should be preferred.

Compared to other DFT-MD methods presented in the literature, it allows for reducing the computational cost while remaining fully ab initio (no classical expression for the magnetic dipole is introduced). The standard DFT-MD methods (eq 1) require computing a number of dipole and magnetic moments that increases linearly with the number of steps and the number of trajectories used. AWV (eq 7) instead scales with the number of explored minima of the Potential Energy Surface, i.e., AWV needs 1–150 APTs and AATs instead of the usual 150 000–500 000 dipole and magnetic moments. One example is the case of the S-PO molecule: AWV makes use only of one single APT and one single AAT tensor for the three trajectories; the standard methods would instead need 150 000 dipole moments and 150 000 magnetic moments.

Moreover, AWV can assign the spectrum to fractions of the system without localization of the wave function or the charge density. Interestingly, AWV uses physical observables, i.e., the APT and the AAT, for partitioning the signal into its molecular components.

AWV shows sensitivity to both intermolecular effects and intermolecular effects. It has the means to distinguish between local intramolecular and intrinsically collective intermolecular modes. Notice also that the AWV method can be easily coupled with methods to build effective normal modes^82^ or graph-theory active modes^83^ from the DFT-MD trajectories and get further insight into the assignment of the vibrational bands.

The high-frequency region (above 2800 cm^–1^) is a more delicate matter than the fingerprint region, and further tests will be required in the future. For example, for the 1S-FEN molecule, the classical nuclei trajectories, selected here as a compromise between accuracy and computational cost, allow only a partial matching with the experimental spectra. Still, using some cautions, a qualitative recognition of the vibrational bands is possible in the high-frequency range. Playing with the temperature to explore a more anharmonic portion of the Potential Energy Surface, one can have an idea of the effects of the activation of mechanical couplings with lower frequency modes. In the future, to improve the match with the experimental spectra also for this region, it would be interesting to couple the AWV method with semiclassical MD simulations^84−86^ that allow, at the price of much higher computational cost, to take into account nuclear quantum effects such as the Zero Point Energy of the vibrational modes.

In any case, it can be concluded that the AWV method generally is a good compromise between accuracy and computational cost for predicting VCD spectra.

## Supporting Information Available

Additional equations for calculating the spectra, additional computational details for the benchmark systems, additional experimental spectra for R2B molecule, and convergence check for the spectra (PDF)
