# Supplementary material for: Vibrational Circular Dichroism from DFT Molecular Dynamics: The AWV Method
Source: J Chem Theory Comput. 2022 Sep 16;18(10):6217–30. doi: 10.1021/acs.jctc.2c00736 (PMC9558311; doi:10.1021/acs.jctc.2c00736)
Supplement: Supplementary file 1 — ct2c00736_si_001.pdf [file ct2c00736_si_001.pdf]

Supporting Information:

Vibrational Circular Dichroism from DFT Molecular Dynamics: the AWV method

Daria Ruth Galimberti[*,a]

[a]Institute for Molecules and Materials, Radboud University, Heyendaalseweg 135, 6525 AJ Nijmegen, The Netherlands

S0. Spectra of individual conformers

AWV provides a simple way to access the spectra of individual conformers once they have been identified from the analysis of the trajectories. In fact, the contribution of a specific conformation to the total signal can be obtained with no additional cost by a modified version of eq 7 of the main text:

$$\Delta I^{conf}(\omega) = \frac{8\pi\beta}{3Vc}\ Im \int dte^{-i\omega t} \langle \sum_{u=x,y,z} \left(\sum_{m}\sum_{i\in m} f_m^{conf}(0) P_{ui}(0) v_i(0)\right)\left(\sum_{n}\sum_{k\in n} f_n^{conf}(t)\ M_{uk}(t) a_k(t)\right)\rangle \quad (S1)$$

where $f_m^{conf}(t)$ and $f_n^{conf}(t)$ are the probabilities that the molecules/fragments m and n are vibrating around the minimum of a specific conformation *conf*.

To evaluate f_m^{conf} (and f_n^{conf}) one can use, for example, the same strategy adopted for evaluating w^j (see section 2.1 of the main text). We can assume a Gaussian distribution around the conformations:[1,2]

$$f_m^{conf} = \exp\left(-\frac{d_{conf}}{2\sigma_c}\right) \Big/ \sum_i \exp\left(-\frac{d_i}{2\sigma_c}\right) \quad (S2)$$

where d_j is a metric measuring the "distance" between the geometry of the molecule/fragment m at instant *t* and the conformation *conf*, and σ_c is the width of the Gaussian.

S1. Five points central difference derivative

Five points central difference formula used in this work to compute the velocities and the accelerations as numerical derivatives of the atomic positions along the trajectory:

$$f'(x) \approx \frac{-f(x+2h) + 8f(x+h) - 8f(x-h) + f(x-2h)}{12h} \quad (S3)$$

$$f''(x) \approx \frac{-f(x+2h) + 16f(x+h) - 30\, f(x) + 16f(x-h) - f(x-2h)}{12h} \quad (S4)$$

S2. (S)-(−)-Propylene oxide computed gas-phase *static* spectra

Figure S1 compares the (S)-(−)-Propylene oxide (S-PO) *static* gas-phase harmonic spectra computed with the BLYP functional and the B3LYP functional (Gaussian16 code.[3], aug-cc-TZvp basis set, Grimme D3 dispersion term[4]).

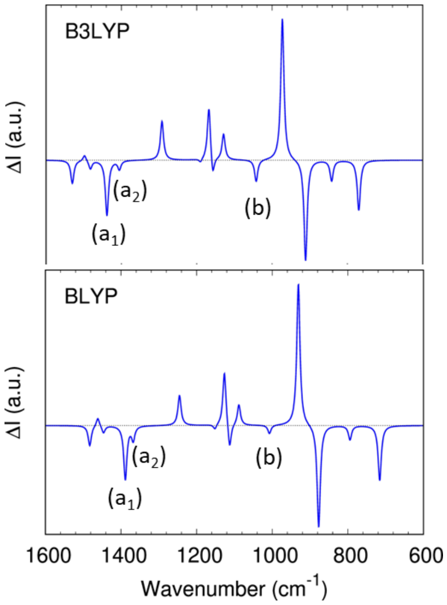

Figure S1. Computed gas-phase *static* VCD spectrum on the isolated (S)-(−)-Propylene oxide molecule in the double harmonic approximation. Top: B3LYP-D3/aug-cc-TZvp. Bottom: BLYP-D3/aug-cc-TZvp.

The main difference between the two descriptions is a general redshift of the bands in the BLYP spectrum compared to the B3LYP one. Also, the negative band around 1020 cm^{-1} (Figure S1- band b) seems somehow underestimated in intensity by the BLYP functional. Finally, the splitting of the two negative bands between 1350 and 1400 cm^{-1} (a1 and a2) is 34 cm^{-1} at the B3LYP level, while it is only 20 cm^{-1} at the BLYP level.

Apart from these, the BLYP functional and B3LYP functional predictions are similar. Therefore, for the DFT-MD simulations on the neat liquid phase of S-PO, the BLYP functional has been preferred to the more expensive B3LYP one.

S3. (S)-(−)-Propylene oxide additional computational details

As a starting point for the DFT-MD simulations, three independent replicas of the system were generated by extracting sets of atomic positions from a classical MD trajectory, each separated from the others by one nanosecond at least. The classical molecular dynamics simulation were carried out with TINKER[5], using the MM3 force field. Both the pre-equilibration (1 ns) and the classical MD production run have been carried out in the NVT ensemble, using the Berendsen thermostat with a time constant of 1 ps. The classical MD simulation consisted of 16 molecules in a cubic box of 12.296 Å x 12.296 Å 12.296 Å. The box sizes were chosen to reproduce the experimental density at room

temperature (0.83 g/cm^3), and periodic boundary conditions were applied in all three spatial directions to mimic a bulk liquid phase.

To predict the time evolution of the Atomic Polar Tensor, **P**, and the Atomic Axial Tensors, **M,** the divide and conquer strategy described in section 2.2 of the main text was applied. Four fragments modelled each molecule (Figure S2): the CH_3 (fragment I), the hydrogens of the CH_2 (fragment II), the hydrogen of the CH (fragment III), and the C-O-C ring (fragment IV).

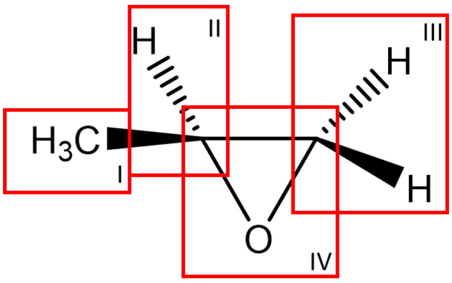

Figure S2. The four fragments used to model each S-PO molecule.

For this simple case, for each molecule, we can define a local metric, $\mathbf{d}_j^{frag}$**,** that is the same for all the fragments of the molecule: the τ_{CH_3} torsional angle describing the CH_3 rotation. Because of the weak interactions between the molecules, it is reasonable to consider that the effect of the intermolecular environment on the tensors of each solvated molecule is small. Therefore, for all four fragments, the required reference structures consisted of a single gas S-PO molecule in different positions along the CH_3 rotational Potential Energy Surface.

Two sets were tested (Table S1). The first (SET A) consists of three reference structures in the *reducible* set (one in the *irreducible* set, see section 2.2 of the main text for the definition) corresponding to the three minima positions on the torsional potential energy surface of the CH_3. The second set (SET B) is also comprehensive of the transition geometries between two minima (maximum of the torsional potential energy surface). This second set consists of two structures in the *irreducible* set and six in the *reducible* one.

Table S1. Number (N ref) of *irreducible* reference structures (i.e., the set without the chemically equivalent minima) and *reducible* reference structures (*irreducible* reference structures) in each of the tested sets for S-PO in the liquid phase.

	Irreducible1 N ref	*Reducible* N ref
SET A	1	2
SET B	3	6

Comparing the spectra computed with the two sets (Figure S3), we can see that using only the three minima positions on the torsional potential energy surface of the CH_3 (SET A), or including transition geometries between two minima (SET B), introduce only negligible differences. Therefore, the additional computational cost of set B is not justified for this particular case. However, the reader should be aware that this is not a general rule; set in other spectral ranges[1] set B can be required.

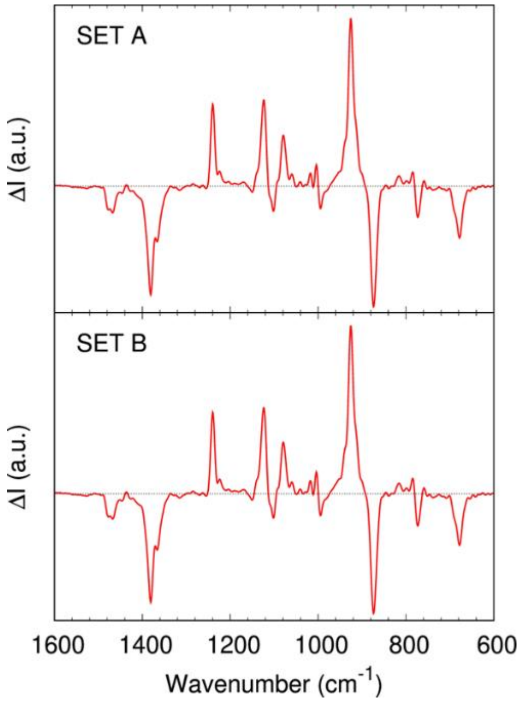

Figure S3. (S)-(−)-Propylene oxide liquid phase theoretical *dynamic* spectrum (AWD method, eq 7 of the main text) using as reference structures only the three minima positions on the torsional potential energy surface of the CH_3 (SET A, top), or including also transition geometries between two minima (SET B, bottom)

S4. (S)-(−)-Propylene oxide experimental spectrum from Ref.[6]

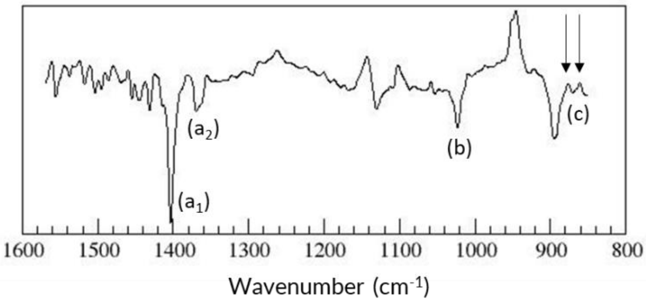

Figure S4. (S)-(−)-Propylene oxide experimental VCD neat liquid phase spectrum, reproduced from Ref. [6].

S5. R-(-)-2butanol additional computational details

As a starting point for the DFT-MD simulations, eight independent replicas of the system were generated by extracting a set of atomic positions from a classical MD trajectory, each separated from the others by one nanosecond at least. The classical molecular dynamics simulation were carried out with TINKER[5], using the OPLS-AA[7] force field. Both the pre-equilibration (1 ns) and the classical MD production run have been carried out in the NVT ensemble, using the Berendsen thermostat with a time constant of 1 ps. The classical MD simulation consists of 32 molecules in a cubic box of 16.956 Å x 16.956 Å 16.956 Å. The box sizes were chosen to reproduce the experimental density at room temperature (0.808 g/cm^3), and periodic boundary conditions were applied in all three spatial directions to mimic a bulk liquid phase.

To predict the time evolution of the Atomic Polar Tensor, **P**, and the Atomic Axial Tensors, **M,** the divide and conquer strategy described in section 2.2 of the main text was applied. Five fragments modelled each molecule (Figure S5): the OH (fragment I), the two CH_3 (fragments II and III), the CH_2 (fragment IV), and the central CH (fragment V).

The following protocol was used to generate the set of reference structures for each fragment. The DFT-MD trajectories were analyzed molecule by molecule, focusing on the intramolecular degrees of freedom, and the explored molecular conformations were classified in terms of intramolecular torsional angles. Subsequently, a set of not-equivalent conformations was selected (*irreducible* set, see section 2.2 of the main text for the definition). For each conformation, a cluster composed of a target R2M molecule with the right torsional angles and all the other R2M molecules H-bonded to it was extracted from the DFT-MD trajectories and used as a model to compute the APT and AAT for the target conformation. This choice (instead of a gas-phase molecule as for the S-PO) is dictated by

the fact that R2B forms strong hydrogen bonds in the liquid phase. The hydrogen bonds have a non-negligible effect on the spectrum; thus, they must be explicitly considered in the calculations of the AAT and APT.

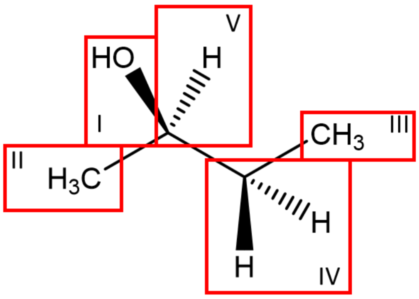

	Metric
OH Frag I	$\{\tau_{OH}\}$
CH_3 Frag II	$\{\tau_{OH}, \tau_{CH_3}^{Frag\ II}\}$
CH_3 Frag III	$\{\tau_{CCCC}, \tau_{CH_3}^{Frag\ III}\}$
CH_2 Frag IV	$\{\tau_{H^{Frag\ IV}CCC}, \tau_{OH}, \tau_{CH_3}^{Frag\ III}\}$
CH Frag V	$\{\tau_{OH}\}$

Figure S5. The five fragments used to model each R2B molecule and the chosen local metrics, $\mathbf{d}_j^{frag}$ (see section 2.1 of the main text for the definition).

Three sets with an increasing number of reference structures and, consequently, computational cost were tested (Table S2).

Table S2. Number (N ref) of *irreducible* reference structures (i.e., the set without the chemically equivalent minima), *reducible* reference structures (*irreducible* reference structures) in each of the tested sets for R2B in the liquid phase.

	Irreducible N ref	*Reducible* N ref
SET A	67	603
SET B	107	963
SET C	612	5508

The first (SET A) consists of 67 reference structures in the *irreducible* set and 603 in the *reducible* one (see section 2.2 of the main text for the definition). The second set (SET B) consists of 107 structures in the *irreducible* set and 963 in the *reducible* one. The third set (SET C) consists of 612 structures in the *irreducible* set and 5508 in the *reducible* one.

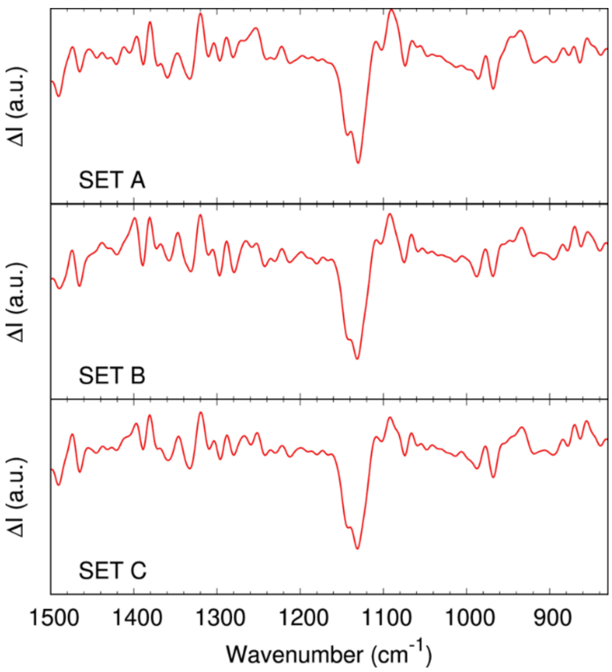

Figure S6. R-(-)-2butanol liquid phase theoretical *dynamic* spectrum (AWD method, eq 7 of the main text) using the three sets described in Table S2.

Figure S6 reports the spectra computed with the three sets. The marker bands characteristic of the R2B spectrum (see the main paper for more details) are correctly described already with the smaller set (SET A). However, SET B guarantees a better description of the region between 1200 cm^{-1} and 1300 cm^{-1}, still at a reasonable computational cost. Instead, the improvement between SET B and SET C is insufficient to justify the six times higher computational cost. Therefore SET B was chosen for the discussion in the main paper.

S6. Convergence check for the R-(-)-2butanol

Figures S7 shows the evolution of total (Dynamic TOT.) and intramolecular (Dynamic INTRA) *dynamic* spectra computed in the fingerprint region with the AWV method (eqs 7 and 9 of the main paper), increasing the number of used independent replicas for the R-(-)-2butanol in the liquid phase. The *dynamic* intramolecular spectrum already converged with only two replicas of the system. Instead, in the case of the total *dynamic* spectrum, significant differences are still visible between four replicas and six replicas.

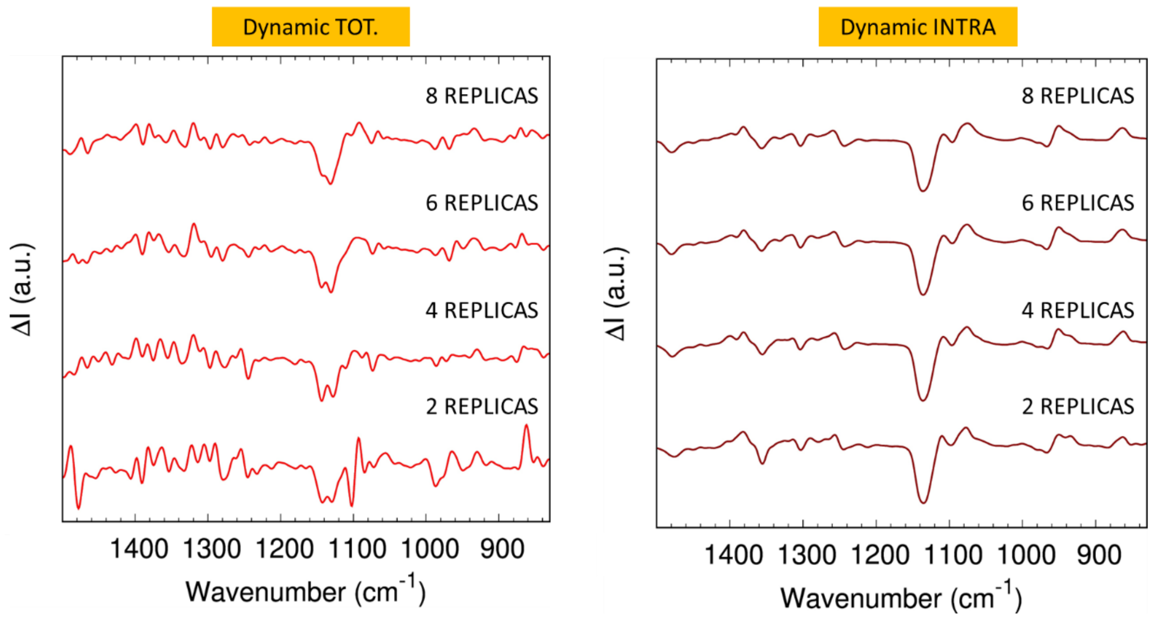

Figure S7. R-(-)-2butanol liquid phase theoretical *dynamic* spectrum (AWV method, eq 7 of the main text) computed using 2, 4, 6, and 8 independent system replicas. Left panel: total spectrum (eq 7 of the main paper). Right panel: intramolecular component of the spectrum (eq 9 of the main paper)

Finally, it is interesting to notice how by increasing the number of replicas, the bands of the region between 1200 and 1400 cm^{-1} broaden, providing a better match with the experimental spectrum. The computational chosen setup (32 molecules in the simulation box and trajectories of 20 ps) cannot completely mimic the heterogeneity of the real liquid. However, we partially compensate for this effect by using independent replicas of the system.

S7. (1S)-Fenchone additional computational details

To predict the time evolution of the Atomic Polar Tensor, **P**, and the Atomic Axial Tensors, **M,** the divide and conquer strategy described in section 2.2 of the main text was applied. Four fragments modelled the (1S)-Fenchone (1S-FEN) molecule in a way to describe the possible CH_3 large amplitude motions (Figure S8): the three CH_3 groups (fragment I, II, and III) and the central body of the molecule (fragment IV).

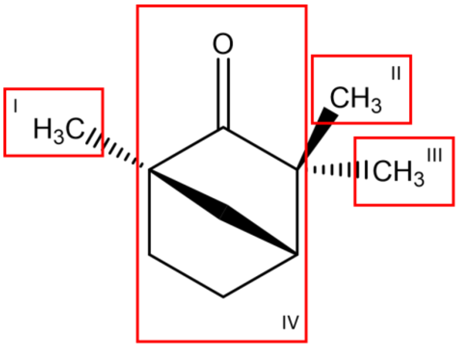

	Metric
CH_3 Frag I	$\{\tau_{CH_3}^{Frag\ I}\}$
CH_3 Frag II	$\{\tau_{CH_3}^{Frag\ II}\}$
CH_3 Frag III	$\{\tau_{CH_3}^{Frag\ III}\}$
Frag IV	$\{\tau_{CH_3}^{Frag\ I}, \tau_{CH_3}^{Frag\ II}, \tau_{CH_3}^{Frag\ III}\}$

Figure S8. The five fragments used to model the 1S-FEN molecule and the chosen local metrics, $\mathbf{d}_j^{frag}$ (see section 2.1 of the main text for the definition).

The required reference structures for all four fragments consisted of a 1S-FEN molecule in different positions along the CH_3 rotational Potential Energy Surface.

Two sets were tested (Table S3). The first (SET A) consists of 27 reference structures in the *reducible* set (3 in the *irreducible* one, see section 2.2 of the main text for the definition) corresponding to the three minima positions on the torsional potential energy surface of the CH_3. The second set (SET B) is also comprehensive of the transition geometries between two minima (maximum of the torsional potential energy surface). This second set consists of six structures in the *irreducible* set and 108 in the *reducible* one.

Table S3. Number (N ref) of *irreducible* reference structures (i.e., the set without the chemically equivalent minima) and *reducible* reference structures (*irreducible* reference structures) in each of the tested sets for 1S-FEN in the gas phase.

	Irreducible N ref	*Reducible* N ref
SET A	3	27
SET B	6	108

If we compare the spectra computed with AWV with the 600 K simulation (600K-*dynamic)* with SET A or SET B, there are almost no differences between the two in the fingerprint region (Figure

S9) and only minor ones in the high-frequency range (Figure S10). At lower temperatures (*dynamic*-50K and *dynamic*-300K spectra), the differences are even more minor because the CH_3 show less large amplitude motions, i.e., fewer contributions of the transition states. Therefore, we can conclude that SET A is enough to predict the spectra in the considered temperature range correctly.

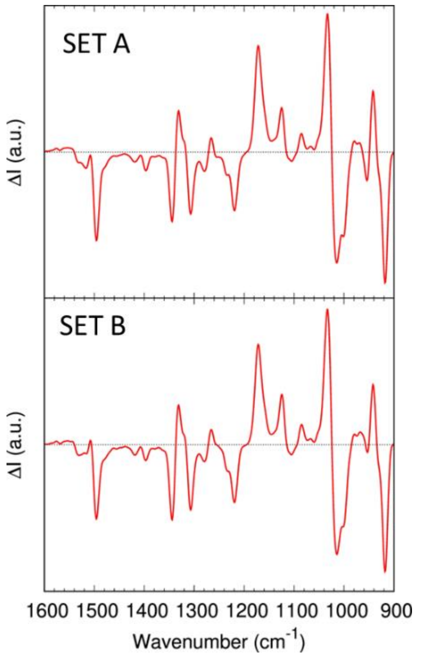

Figure S9. (1S)-Fenchone gas-phase theoretical *dynamic* spectrum (AWV method, eq 7 of the main text) in the fingerprint region using the two sets of reference structures described in Table S3

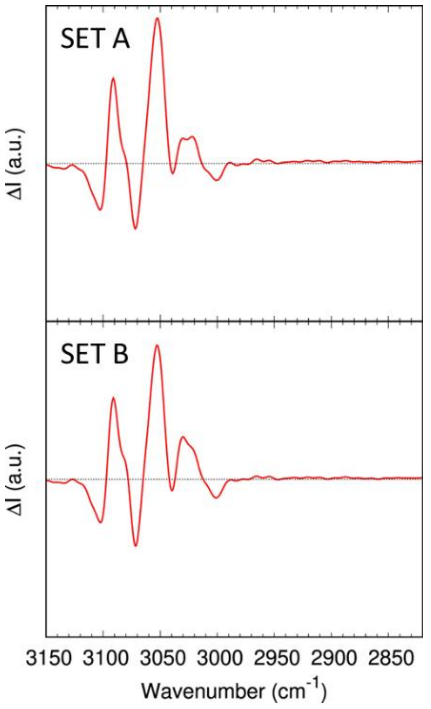

Figure S10. (1S)-Fenchone gas-phase theoretical *dynamic* spectrum (AWV method, eq 7 of the main text) in the high-frequency range using the two sets of reference structures described in Table S3

S8. Convergence check for the (1S)-Fenchone

Figures S11, S12, and S13 show the evolution of the gas-phase (1S)-Fenchone predicted *dynamic* spectra (AWV method, eq 7 of the main paper), increasing the number of used independent replicas when the simulations are run at 50 K, 300 K, and 600 K.

In general, increasing the temperature, a more significant portion of the potential energy surface is accessible for the system; therefore, an increased number of replicas is required to have a correct sampling. The tests show that the high-frequency region (above 2800 cm^{-1}) is more sensitive to sampling issues and requires a larger number of replicas to converge the spectra compared to the fingerprint region. We can probably relate this to the strong mechanical coupling of the CH_3 torsions with the CH-stretching. Interestingly, the symmetric stretching modes appear more sensitive to the coupling than anti-symmetric ones (see section 3.3.3 of the main paper for more details).

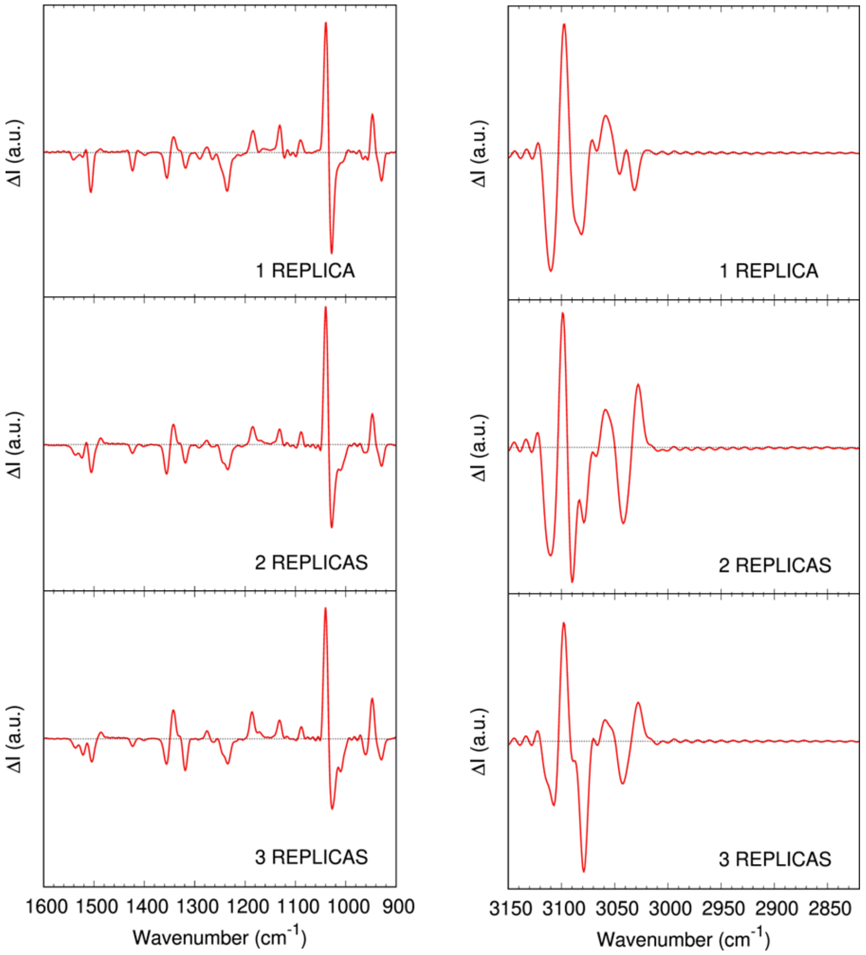

Figure S11. (1S)-Fenchone gas-phase theoretical *dynamic* spectrum (AWV method, eq 7 of the main text) computed at 50 K using 1, 2, and 8 independent replicas of the system. Left panel: fingerprint region. Right panel: high-frequency region.

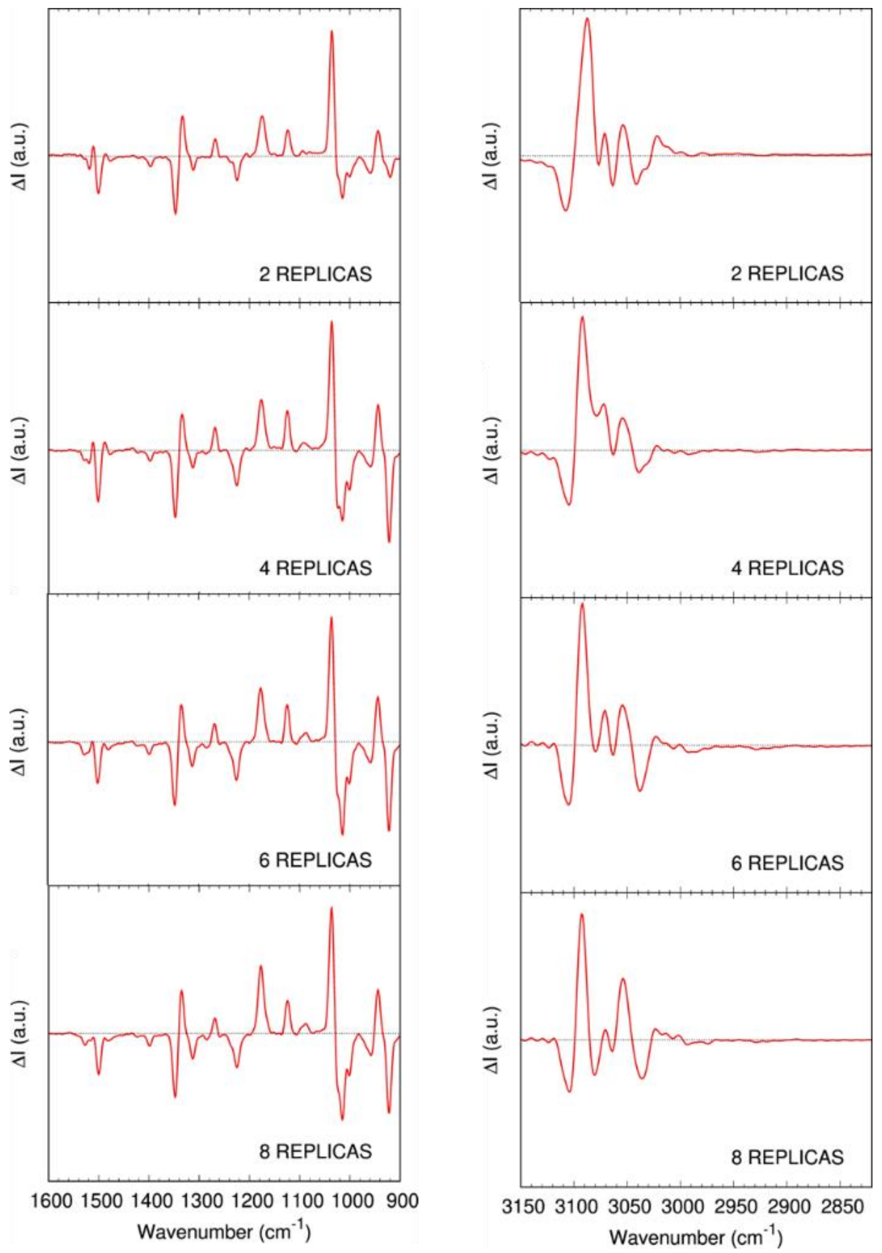

Figure S12. (1S)-Fenchone gas-phase theoretical *dynamic* spectrum (AWV method, eq 7 of the main text) computed at 300 K using 2, 4, 6, and 8 independent system replicas. Left panel: fingerprint region. Right panel: high-frequency region.

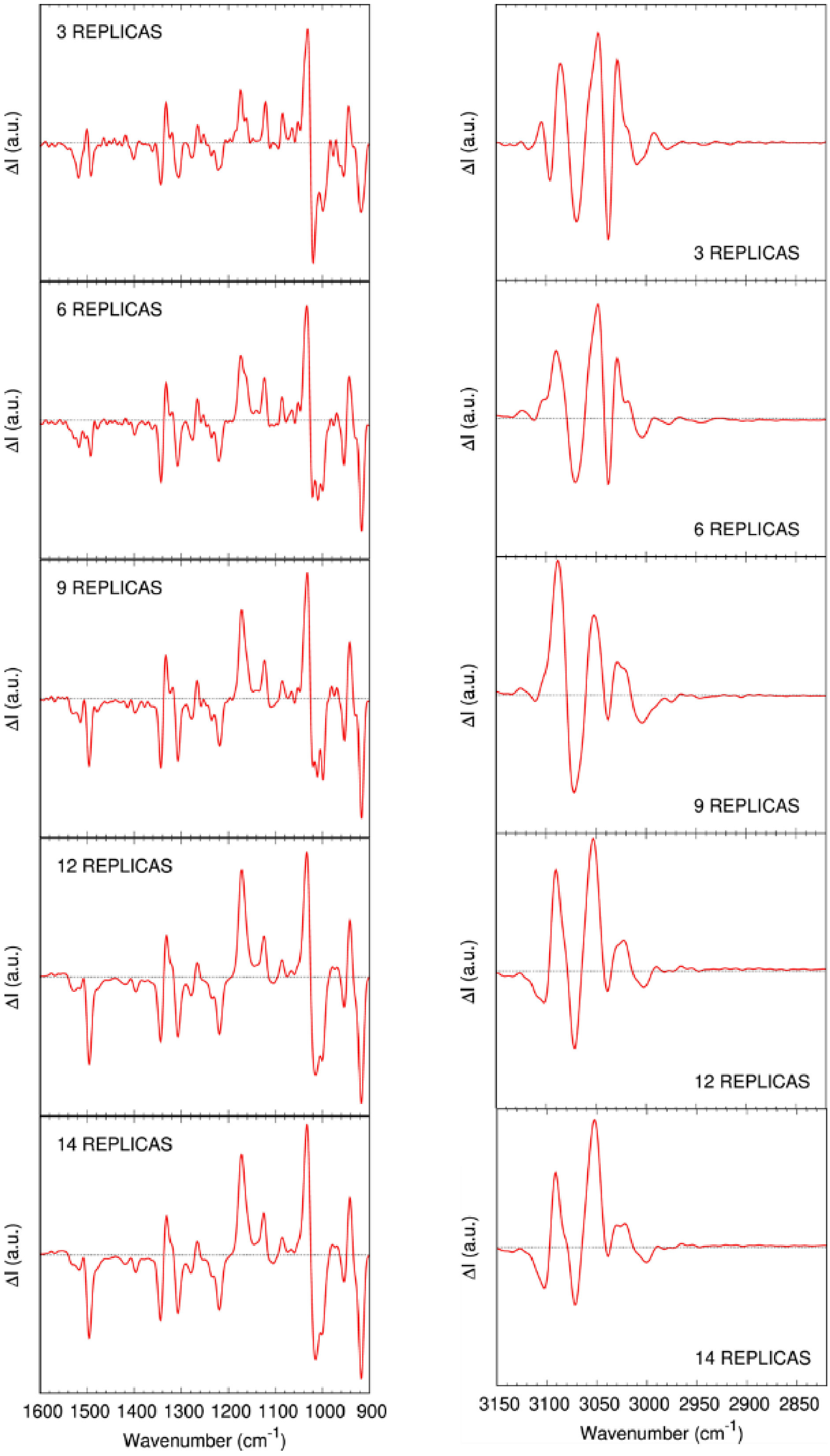

Figure S13. (1S)-Fenchone gas-phase theoretical *dynamic* spectrum (AWV method, eq 7 of the main text) computed at 600 K using 3, 6, 9, 12, and 14 independent system replicas. Left panel: fingerprint region. Right panel: high-frequency region.

References:

1. Galimberti, D. R.; Milani, A.; Tommasini, M.; Castiglioni, C.; Gaigeot, M.-P., Combining Static and Dynamical Approaches for Infrared Spectra Calculations of Gas Phase Molecules and Clusters. *J. Chem. Theory Comput.* **2017,** *13*, 3802-3813, https://doi.org/10.1021/acs.jctc.7b00471.

2. Mathias, G.; Ivanov, S. D.; Witt, A.; Baer, M. D.; Marx, D., Infrared Spectroscopy of Fluxional Molecules from (ab Initio) Molecular Dynamics: Resolving Large-Amplitude Motion, Multiple Conformations, and Permutational Symmetries. *J. Chem. Theory Comput.* **2012,** *8*, 224-234, https://doi.org/10.1021/ct2006665.

3. Frisch, M. J.; Trucks, G. W.; Schlegel, H. B.; Scuseria, G. E.; Robb, M. A.; Cheeseman, J. R.; Scalmani, G.; Barone, V.; Petersson, G. A.; Nakatsuji, H.; Li, X.; Caricato, M.; Marenich, A. V.; Bloino, J.; Janesko, B. G.; Gomperts, R.; Mennucci, B.; Hratchian, H. P.; Ortiz, J. V.; Izmaylov, A. F.; Sonnenberg, J. L.; Williams; Ding, F.; Lipparini, F.; Egidi, F.; Goings, J.; Peng, B.; Petrone, A.; Henderson, T.; Ranasinghe, D.; Zakrzewski, V. G.; Gao, J.; Rega, N.; Zheng, G.; Liang, W.; Hada, M.; Ehara, M.; Toyota, K.; Fukuda, R.; Hasegawa, J.; Ishida, M.; Nakajima, T.; Honda, Y.; Kitao, O.; Nakai, H.; Vreven, T.; Throssell, K.; Montgomery Jr., J. A.; Peralta, J. E.; Ogliaro, F.; Bearpark, M. J.; Heyd, J. J.; Brothers, E. N.; Kudin, K. N.; Staroverov, V. N.; Keith, T. A.; Kobayashi, R.; Normand, J.; Raghavachari, K.; Rendell, A. P.; Burant, J. C.; Iyengar, S. S.; Tomasi, J.; Cossi, M.; Millam, J. M.; Klene, M.; Adamo, C.; Cammi, R.; Ochterski, J. W.; Martin, R. L.; Morokuma, K.; Farkas, O.; Foresman, J. B.; Fox, D. J. *Gaussian 16 Rev. C.01*, Wallingford, CT, 2016.

4. Grimme, S.; Antony, J.; Ehrlich, S.; Krieg, H., A consistent and accurate ab initio parametrization of density functional dispersion correction (DFT-D) for the 94 elements H-Pu. *J. Chem. Phys.* **2010,** *132*, 154104, https://doi.org/10.1063/1.3382344.

5. Rackers, J. A.; Wang, Z.; Lu, C.; Laury, M. L.; Lagardère, L.; Schnieders, M. J.; Piquemal, J.-P.; Ren, P.; Ponder, J. W., Tinker 8: Software Tools for Molecular Design. *J. Chem. Theory Comput.* **2018,** *14*, 5273-5289, https://doi.org/10.1021/acs.jctc.8b00529.

6. Polavarapu, P. L.; Michalska, D. F., Vibrational circular dichroism in (S)-(-)-epoxypropane. Measurement in vapor phase and verification of the perturbed degenerate mode theory. *J. Am. Chem. Soc.* **1983,** *105*, 6190-6191, https://doi.org/10.1021/ja00357a059.

7. Jorgensen, W. L.; Maxwell, D. S.; Tirado-Rives, J., Development and Testing of the OPLS All-Atom Force Field on Conformational Energetics and Properties of Organic Liquids. *J. Am. Chem. Soc.* **1996,** *118*, 11225-11236, https://doi.org/10.1021/ja9621760.
